# Loss of MTSS1 results in increased metastatic potential in pancreatic cancer

**DOI:** 10.18632/oncotarget.14869

**Published:** 2017-01-27

**Authors:** Ann E Zeleniak, Wei Huang, Mary K Brinkman, Melissa L Fishel, Reginald Hill

**Affiliations:** ^1^ Integrated Biomedical Sciences Program, University of Notre Dame, South Bend, Indiana, USA; ^2^ Harper Cancer Research Institute, University of Notre Dame, South Bend, Indiana, USA; ^3^ Department of Biological Sciences, University of Notre Dame, South Bend, Indiana, USA; ^4^ Indiana University School of Medicine, Department of Pharmacology and Toxicology, Indianapolis, Indiana, USA; ^5^ Indiana University School of Medicine, Department of Pediatrics, Wells Center for Pediatric Research, Indianapolis, Indiana, USA; ^6^ Pancreatic Cancer Signature Center, Indianapolis, Indiana, USA

**Keywords:** pancreatic cancer, metastasis, MTSS1, inflammation, tumor microenvironment

## Abstract

Pancreatic ductal adenocarcinoma (PDAC) has a 5-year survival rate of 7%. This dismal prognosis is largely due to the inability to diagnose the disease before metastasis occurs. Tumor cell dissemination occurs early in PDAC. While it is known that inflammation facilitates this process, the underlying mechanisms responsible for this progression have not been fully characterized. Here, we functionally test the role of metastasis suppressor 1 (MTSS1) in PDAC. Despite evidence showing that MTSS1 could be important for regulating metastasis in many different cancers, its function in PDAC has not been studied. Here, we show that loss of MTSS1 leads to increased invasion and migration in PDAC cell lines. Moreover, PDAC cells treated with cancer-associated fibroblast-conditioned media also have increased metastatic potential, which is augmented by loss of MTSS1. Finally, overexpression of MTSS1 in PDAC cell lines leads to a loss of migratory potential *in vitro* and an increase in overall survival *in vivo*. Collectively, our data provide insight into an important role for MTSS1 in suppressing tumor cell invasion and migration driven by the tumor microenvironment and suggest that therapeutic strategies aimed at increasing MTSS1 levels may effectively slow the development of metastatic lesions, increasing survival of patients with PDAC.

## INTRODUCTION

Pancreatic ductal adenocarcinoma (PDAC) is now the 3^rd^ leading cause of cancer-related deaths in the United States [1, 2] with a 5-year survival rate of just 7% [1]. Furthermore, by 2030, PDAC is projected to surpass colorectal cancer to become the second leading cause of cancer-related deaths [3]. This dismal outlook is largely due to the inability to diagnose the disease before metastasis occurs. 53% of patients afflicted with pancreatic cancer are diagnosed at the metastatic stage [4]. Since few patients have resectable disease at the time of diagnosis, these patients are offered either treatment regimens that are unsuccessful [5, 6] or palliative care to ease their pain. Thus, there is a critical need to better understand what causes early tumor cell dissemination and metastatic progression in this disease.

One of the hallmarks of early stages of PDAC is inflammation [7, 8]. It is well known that patients with chronic pancreatitis have a much higher chance to develop PDAC [9–12]. While many studies have focused on elucidating the mechanisms by which this inflammation drives PDAC progression, few have focused on the role inflammation plays in metastasis. Cancer-associated fibroblasts (CAFs) are one of the major components of the inflammatory tumor microenvironment that is a hallmark of PDAC. CAFs make up the majority of the cells that comprise the tumor bulk in PDAC [6, 13, 14]. While many studies investigating how CAFs influence PDAC initiation and progression have been performed [13, 15–17], how CAFs influence the PDAC metastatic cascade is only beginning to be uncovered [18], leaving this area of research largely an enigma [19].

We previously established a mouse model of PDAC where tumor development is driven by a mutant allele of *Kras* and *Pten* heterozygosity [20]. Subsequently, we set out to determine how inflammation contributes to tumor development. In order to elucidate this, we overexpressed cyclooxygenase-2 (COX-2) in our mouse model of PDAC. Cyclooxygenases, COX-1 and COX-2 are enzymes that are essential for production of prostaglandins [21]. While COX-1 is a constitutively expressed housekeeping enzyme, COX-2 expression is upregulated in pancreatitis [22] and pancreatic cancer [23]. We previously tested how *Cox-2* overexpression would affect tumorigenesis in the *Kras/Pten* mouse model of PDAC. Our data showed that *Cox-2* overexpression leads to not only accelerated PDAC tumor development, but also dense tumor stroma formation [24].

Studies show that COX-2 overexpression is positively correlated with increased tumorigenic and metastatic potential in breast [25], gastric [26], and colon cancer [27]. These results suggest that the inflammation driven by COX-2 expression plays an important role in tumor cell dissemination and metastasis. However, the mechanisms through which COX-2 overexpression causes increased metastasis require further elucidation. In this study, starting from our mouse model of PDAC, we show that inflammation in PDAC is correlated with loss of a recently discovered metastatic tumor suppressor gene, metastasis suppressor 1 (MTSS1). Moreover, we show that CAF-derived factors are capable of decreasing the expression level of MTSS1. Furthermore, PDAC cells lacking MTSS1 expression have a more invasive and migratory phenotype, whereas overexpression of MTSS1 significantly reduces these metastatic characteristics. Finally, we show that overexpression of MTSS1 in metastatic PDAC cell lines leads to an increase in overall survival *in vivo*, suggesting that MTSS1 levels may play a critical role in regulating the metastatic cascade of PDAC.

## RESULTS

### Mouse/human Affymetrix Array analysis comparison identifies MTSS1 as inflammation-mediated gene that correlates with patient prognosis

**Figure 1:**
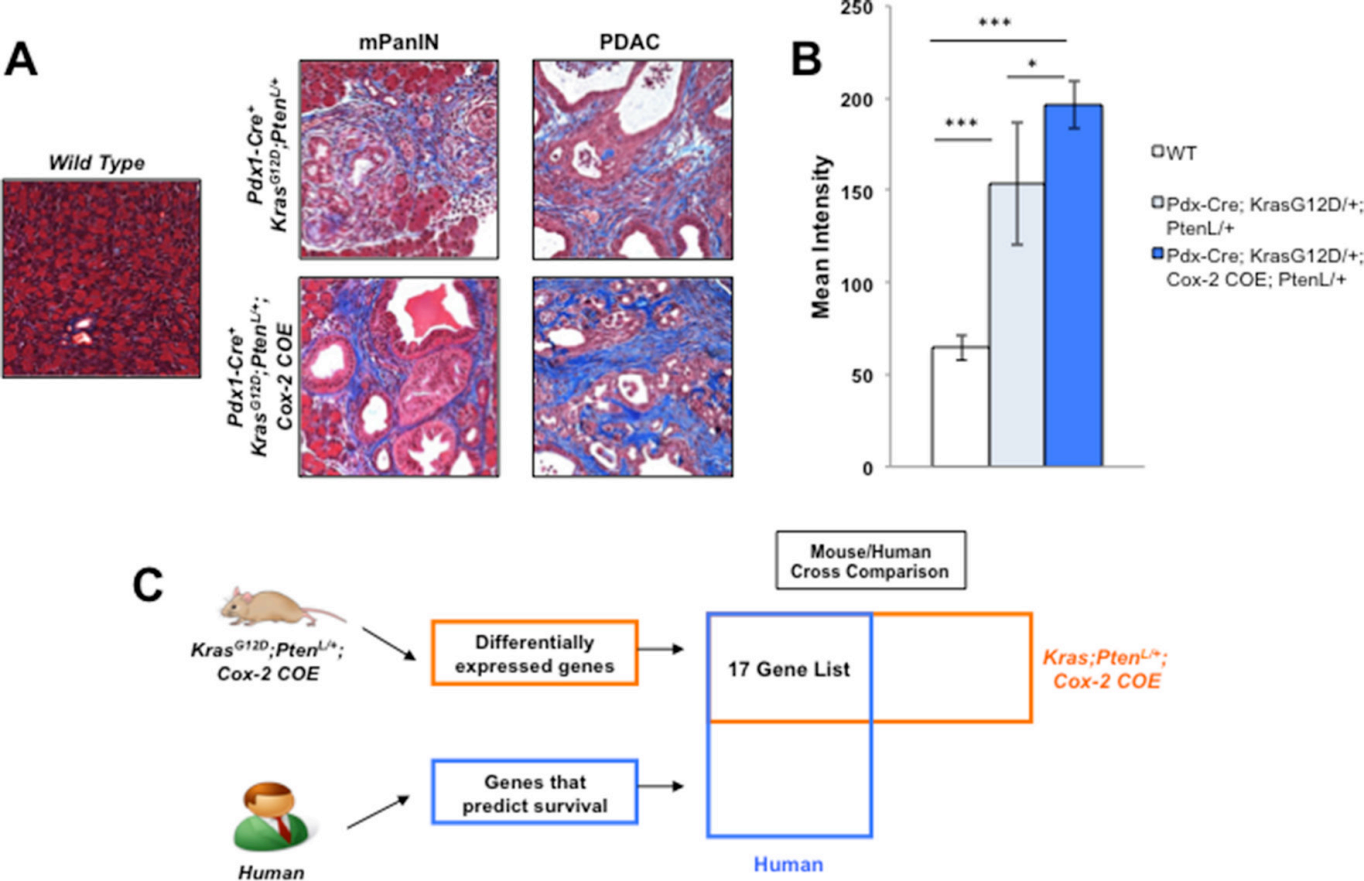
Mouse/Human array data comparison identifies MTSS1 as an inflammation linked gene that predicts poor prognosis in human PDAC (**A**) Masson's trichrome staining for reactive stroma presence in samples from wild type mice, *Kras^G12D/+^;Pten^lox/+^* mice, and *Kras^G12D/+^;Pten^lox/+^;Cox-2* COE mice. (**B**) Quantification of staining intensity of Masson's trichrome staining of tissue from wild type mice, *Kras^G12D/+^;Pten^lox/+^* mice, and *Kras^G12D/+^;Pten^lox/+^;Cox-2* COE mice. (**C**) Schematic detailing strategy for comparing mouse and human array data in order to obtain a list of genes that are differentially expressed in *Kras^G12D/+^;Pten^lox/+^;Cox-2* COE mice that also predict survival in human PDAC patients. *p-value < 0.05, ***p-value < 0.0001.

In order to investigate how the dense tumor microenvironment affects PDAC metastasis, we utilized a genetically engineered mouse model that exhibits severe desmoplasia to determine which proteins are altered under inflammatory conditions. *Kras*^G12D/+^*;Pten*^lox/+^*;Cox-2 COE* mice displayed more intense Trichrome staining in both the PanIN and PDAC stage as compared to the *Kras*^G12D/+^*;Pten*^lox/+^ mice and wild type mice (Figure 1A, 1B). These results indicated that inflammation mediated by COX-2 expression leads to a significant increase in reactive stroma during tumor progression.

We then investigated the genomic profile of the *Kras*^G12D/+^*;Pten*^lox/+^*;Cox-2 COE* mice via Affymetrix Array analysis. Our previous Affymetrix Array analysis identified genes differentially expressed in *Kras*^G12D/+^*;Pten*^lox/+^*;Cox-2 COE* mice compared to non-tumor controls [24] (GSE38988). We took those differentially expressed genes and compared them to a list of genes indicative of poor prognosis identified in an Affymetrix analysis of human PDAC patient samples [28] (GSE32688) in order to identify candidate genes that linked inflammation and poor prognosis (Figure 1C). 17 genes differentially expressed in *Kras*^G12D/+^*;Pten*^lox/+^*;Cox-2 COE* mice that also were on the list of genes indicative of poor prognosis in PDAC patients were identified from this mouse/human comparison (Supplemental Table 1). Expression of the metastatic tumor suppressor gene, metastasis suppressor 1 (MTSS1), was decreased (2.46-fold) in the *Kras*^G12D/+^*;Pten*^lox/+^*;Cox-2 COE* mice compared to baseline of the 17 genes on our list (Supplemental Table 1). Moreover, MTSS1 was a gene from our list that had been previously linked to metastatic progression in a number of different cancer models [29, 33], but that had yet to be investigated in pancreatic cancer. Thus, we chose to focus our subsequent analysis on MTSS1.

### MTSS1 expression correlates with metastatic potential of human PDAC cell lines

In order to determine if MTSS1 expression correlated with metastatic potential, we determined the level of MTSS1 expression in six human pancreatic cancer cell lines that were originally derived from either primary or metastatic lesions. PANC-1, MIA PaCa-2, and BxPC-3 cells are derived from primary pancreatic cancer sites [34], whereas L3.6pl, Hs 766T, and AsPC-1 cells were all derived from pancreatic cancer metastatic sites [34, 35]. Western blot analysis showed that the three PDAC cell lines derived from primary lesions display higher MTSS1 expression levels overall compared to PDAC cell lines derived from metastatic lesions (Supplementary Figure 1A, 1B).

### Loss of MTSS1 leads to a more invasive and migratory phenotype in PDAC cells

**Figure 2:**
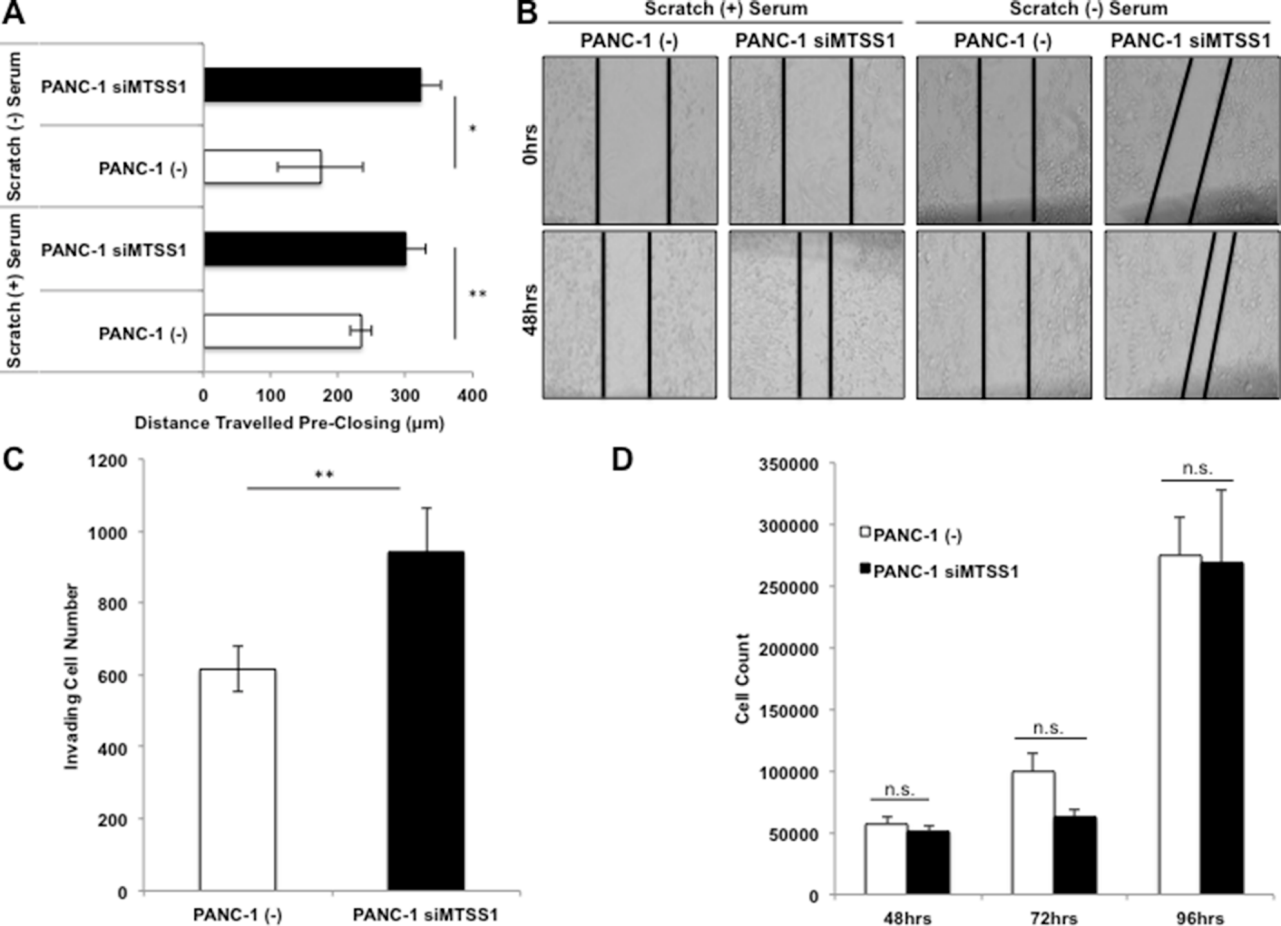
Loss of MTSS1 leads to a more invasive and migratory, but not more proliferative phenotype in PDAC (**A**) Scratch assays were performed in both serum-containing and serum-free conditions with PANC-1 (–) and PANC-1 siMTSS1 cells. (**B**) Representative scratch assay images of PANC-1 (–) and PANC-1 siMTSS1 cells in either the presence or absence of serum. Images were taken at 10X magnification. (**C**) PANC-1 (–) and PANC-1 siMTSS1 cells were plated for a transwell migration assay in the presence of Matrigel, stained with hematoxylin, and counted after 48 hours of incubation. (**D**) PANC-1 (–) and PANC-1 siMTSS1 cells were plated and harvested at various timepoints to determine any changes in proliferative abilities. *p-value < 0.05, **p-value < 0.001.

In order to elucidate the effect that loss of MTSS1 has on cells derived from primary PDAC sites, cell invasion and migration assays were performed on PANC-1 cells. PANC-1 cells, which were found to express a moderate level of MTSS1 (Supplementary Figure 1A, 1B), were treated with either (–) control siRNA or MTSS1 siRNA (siMTSS1) to establish a transient knockdown of MTSS1 expression. Knockdown of MTSS1 was confirmed via RT-qPCR and western blot analysis (Supplementary Figure 2A–2C). We attained 50% knockdown of MTSS1 expression using a siMTSS1 combination compared to (–) control siRNA (Supplementary Figure 2A–2C). Compared to (–) control, siMTSS1 PANC-1 cells exhibit significantly increased migration via scratch assay analyses (Figure 2A, representative images, Figure 2B). Knockdown of MTSS1 causes PANC-1 cells to migrate approximately 150μm farther in serum-free conditions and 50μm farther in wound healing serum-containing conditions over a 48-hour period (Figure 2A). Next, (–) control and siMTSS1 PANC-1 cells were plated on Matrigel-coated transwell membranes in order to determine if MTSS1 knockdown had any effect on tumor cell invasion. PANC-1 cells treated with siMTSS1 show a significant 1.67-fold increase in the ability to invade through the Matrigel-coated membrane compared to PANC-1 cells treated with (–) control siRNA (Figure 2C, representative images, Supplementary Figure 3).

These experiments were performed in an additional PDAC cell line derived from a primary site, MIA PaCa-2 cells. MTSS1 was transiently knocked down using siRNA in MIA PaCa-2 cells (Supplementary Figure 4A, 4B). Control and siMTSS1 MIA PaCa-2 cells were then plated in serum-containing and serum-free scratch assay conditions. It was found that siMTSS1 MIA PaCa-2 cells were significantly more capable of migrating in serum-containing conditions, travelling over 100 μm farther than control as well as in serum-free conditions, travelling over 100μm farther than control (Supplementary Figure 4C). Additionally, siMTSS1 MIA PaCa-2 cells were able to migrate over 3-fold farther than (–) control (Supplementary Figure 4D).

Our data thus far showed that knockdown of MTSS1 promoted cell migration and invasion. We next investigated if MTSS1 played a role in cell proliferation. Our data show that there is no significant difference in proliferation between (–) control and siMTSS1 PANC-1 cells over a 96-hour period (Figure 2D). These results indicate that MTSS1 knockdown significantly increases PDAC cell migration and invasion, but it has no effect on cell proliferation.

### Overexpression of MTSS1 leads to a less invasive and migratory PDAC phenotype

**Figure 3:**
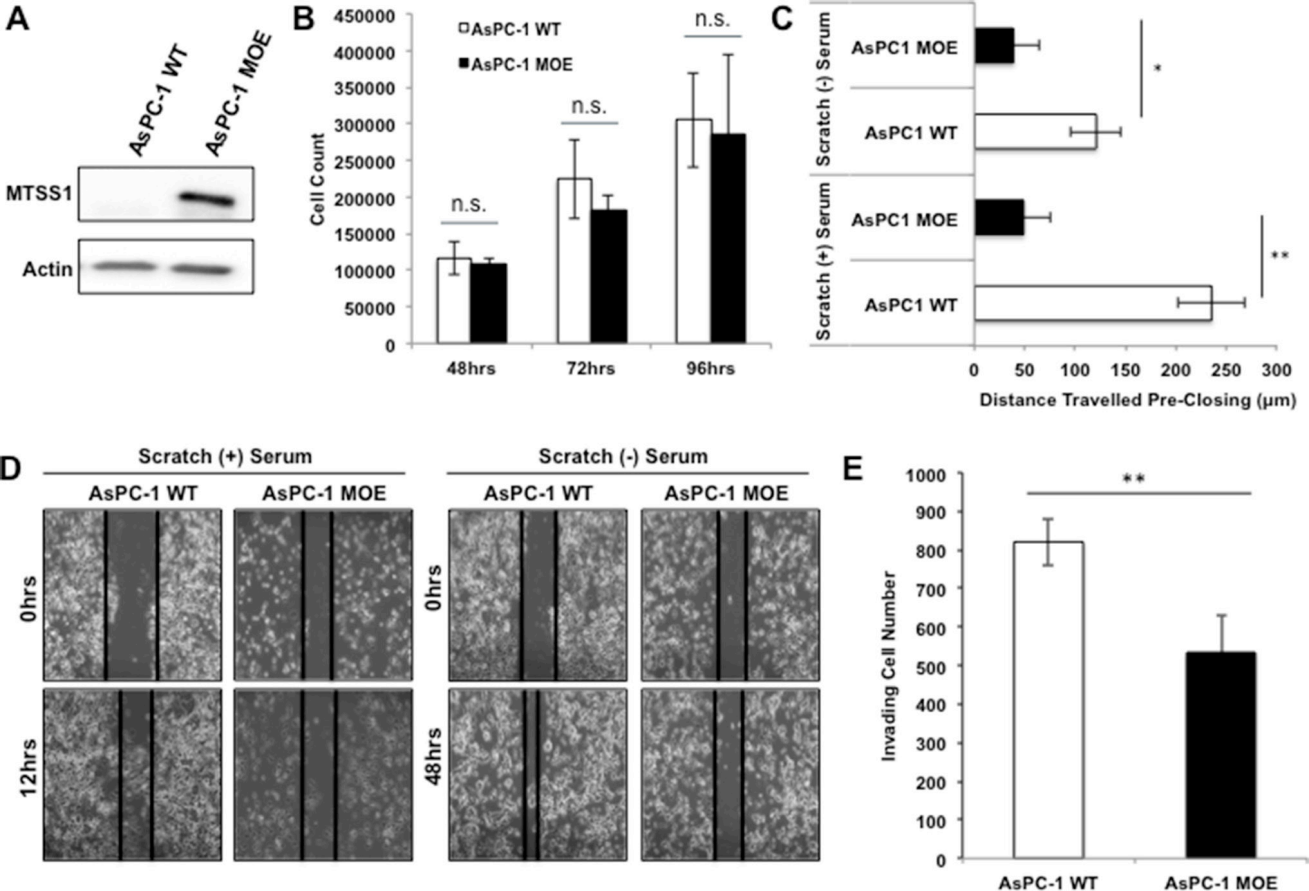
Overexpression of MTSS1 leads to a less invasive and migratory PDAC phenotype (**A**) AsPC-1 metastatic PDAC cells were transduced with MTSS1 plasmid to establish a transient overexpression of MTSS1. (**B**) AsPC-1 (–) and AsPC-1 MOE cells were plated and harvested at various timepoints to determine any changes in proliferative abilities. (**C**) Scratch assays were performed in both serum-containing and serum-free conditions with AsPC-1 WT and AsPC-1 MOE cells. (**D**) Representative scratch assay images of AsPC-1 WT and AsPC-1 MOE cells in either the presence or absence of serum. Images were taken at 10X magnification. (**E**) AsPC-1 WT and AsPC-1 MOE cells were plated for a transwell migration assay in the presence of Matrigel, stained with hematoxylin, and counted after 48 hours of incubation. *p-value < 0.05, **p-value < 0.001.

Having established that loss of MTSS1 leads to a more invasive and migratory phenotype in PDAC cells derived from primary tumor sites, we next overexpressed MTSS1 in a PDAC cell line derived from a metastatic site (AsPC-1) in order to investigate the effect it would have on cell invasion and migration. AsPC-1 cells were chosen because of their lack of MTSS1 expression (Supplementary Figure 1A, 1B). AsPC-1 cells transfected with a MTSS1 plasmid (AsPC-1 MOE) display increased protein expression of MTSS1 compared to control cells (Figure 3A). Similar to the results seen in the proliferation assays between PANC-1 (–) control cells versus siMTSS1 cells, there was no significant difference seen in the proliferative ability of AsPC-1 WT cells as compared to AsPC-1 MOE cells (Figure 3B). However, AsPC-1 MOE cells plated at the same cellular density as control (200,000 cells/well) display significantly decreased migration in both serum-containing scratch assays (∼4-fold) and serum-free scratch assays (∼2-fold) as compared to AsPC-1 WT control (Figure 3C, representative images, Figure 3D). Furthermore, AsPC-1 MOE cells exhibit a significant 1.6-fold decrease in the ability to invade through the Matrigel-coated membrane compared to AsPC-1 WT cells (Figure 3E, representative images, Supplementary Figure 5). These results show that MTSS1 overexpression in metastatic cells with low MTSS1 expression leads to significantly decreased cell migration and invasion, but has no effect on proliferation. These functional assays were also completed in a second cell line, namely PANC-1 cells, through the same transfection method used for the AsPC-1 cell overexpression (Supplementary Figure 6A, 6B). PANC-1 cells that overexpress MTSS1 were significantly less migratory in both serum-free and serum-containing conditions, with the cells migrating approximately 50 μm and 75 μm less, respectively (Supplementary Figure 6C). Additionally, PANC-1 MOE cells were significantly less able (∼1.8-fold) to migrate through a Matrigel-coated membrane as compared to PANC-1 WT cells (Supplementary Figure 6D).

### CAF-conditioned media decreases MTSS1 expression and increases migration, invasion, and proliferation of PDAC cells

**Figure 4:**
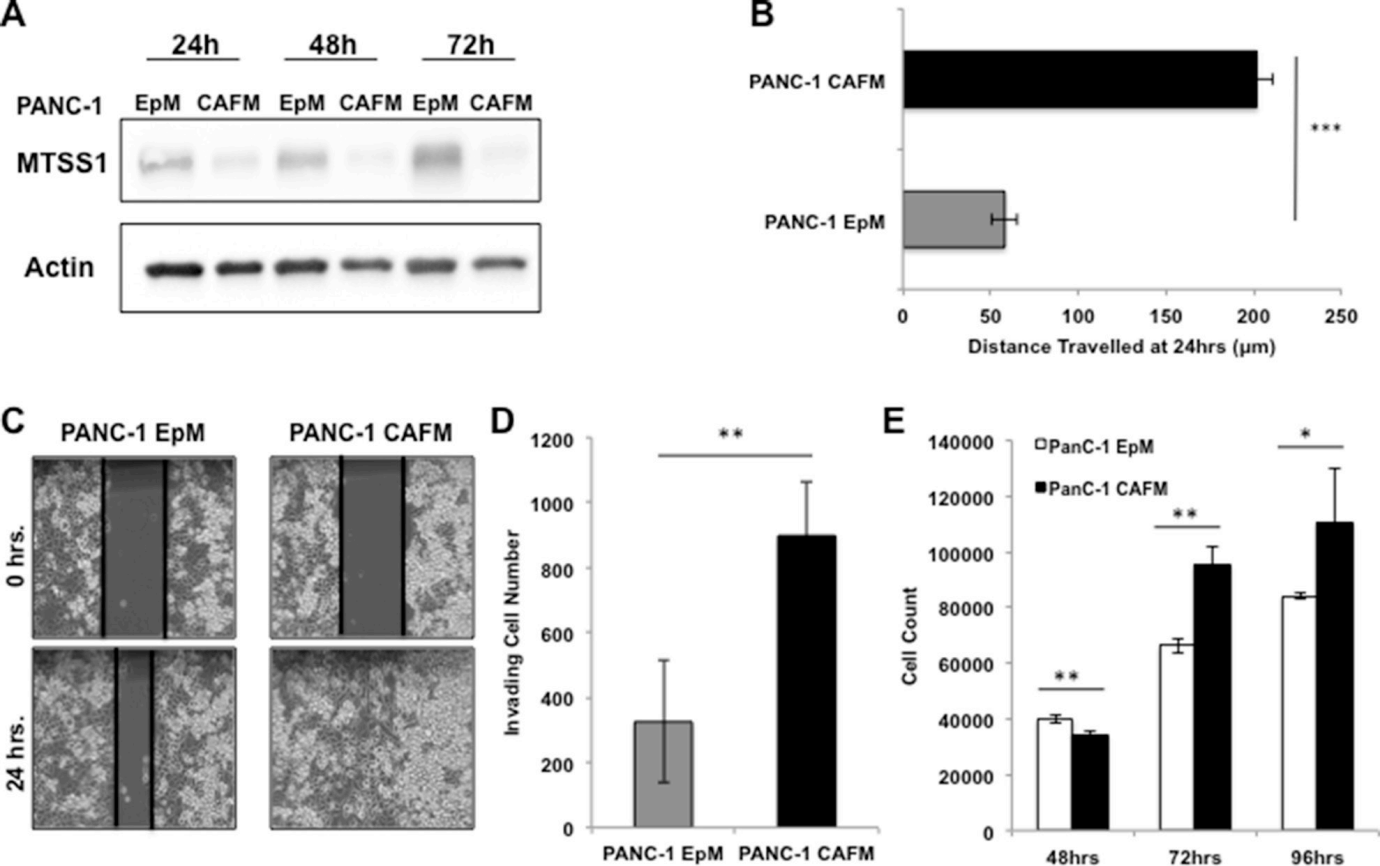
Treatment with CAF media results in decreased MTSS1 and increased proliferation and migration in PANC-1 cells (**A**) Western blot analysis of PANC-1 cells treated with either epithelial-conditioned media (EpM) or CAF-conditioned media (CAFM) and harvested at various timepoints. (**B**) PANC-1 cells were treated with either EpM or CAFM and submitted to a scratch assay for 24 hours. (**C**) Representative images of PANC-1 cells treated with either EpM or CAFM during scratch assay analysis. Images were taken at 10× magnification. (**D**) PANC-1 cells were plated for a transwell migration assay with either EpM or CAFM in the upper chamber. After 48 hours, cells were fixed to the membrane, stained with hematoxylin, and counted for analysis. (**E**) PANC-1 cells were incubated in either CAFM or EpM and harvested at various timepoints to observe any changes in proliferative ability. *p-value < 0.05, **p-value < 0.001, ***p-value < 0.0001.

Thus far, our data show that cell-intrinsic expression of MTSS1 affects cell migration and invasion in PDAC cells. We had originally identified MTSS1 while searching for metastasis-related genes whose expression was linked to COX-2-driven desmoplasia (Figure 1). Thus, we next sought out to determine if cell-extrinsic factors from fibroblast cells played a role in MTSS1 expression and the invasion and migration of PDAC cells. In order to explore how the fibroblasts impact metastatic potential in PDAC, we first treated PANC-1 cells with conditioned media from PDAC patient-derived cancer-associated fibroblast (CAF) cells. These CAF-conditioned media (CAFM) cells were compared via western blot analysis to PANC-1 epithelial-conditioned media (EpM) at various timepoints to determine any changes CAF exposure has on PANC-1 cells. Our data show that PANC-1 cells treated with CAFM had lower expression of MTSS1 at any lysate harvest timepoint (Figure 4A). Additionally, PANC-1 cells were treated with fresh, non-conditioned media (FreshM), PANC-1 EpM, or CAFM for 48 hours. Our data show that PANC-1 cells treated with CAF-conditioned media exhibit increased COX-2 expression as well as 3-fold lower MTSS1 expression at 48 hours when compared to both non-conditioned and epithelial-conditioned media (Supplementary Figure 7A, 7B).

Next, we performed a variety of assays to determine if CAF-conditioned media affects invasion and migration of PDAC cells. PANC-1 cells were plated and, upon reaching confluence, were scratched and incubated in either EpM or CAFM conditions. CAF media-treated cells (PANC-1 CAFM) migrate significantly farther (∼4-fold) than epithelial media-treated cells (PANC-1 EpM) at just 24 hours (Figure 4B, representative images, Figure 4C). PANC-1 cells were then utilized for a transwell invasion assay, where they were either incubated in EpM or CAFM conditions. Results show that there is a significant 2.5-fold increase in the number of cells able to migrate across the membrane in the PANC-1 cells treated with CAFM compared to PANC-1 cells treated with EpM (Figure 4D, representative images, Supplementary Figure 8).

Finally, we investigated whether CAF-conditioned media affects proliferation of PDAC cells. PANC-1 cells were incubated in either CAFM or EpM and harvested at various timepoints to observe any change in proliferative ability. Our data show that PANC-1 cells treated with CAFM are significantly more proliferative than PANC-1 cells treated with EpM (Figure 4E). These results show that CAF-conditioned media has the ability to decrease MTSS1 expression and increase the migration, invasion, and proliferation of PDAC cells.

### Knockdown of MTSS1 augments increased metastatic phenotype seen in PDAC cells treated with CAF-conditioned media

**Figure 5:**
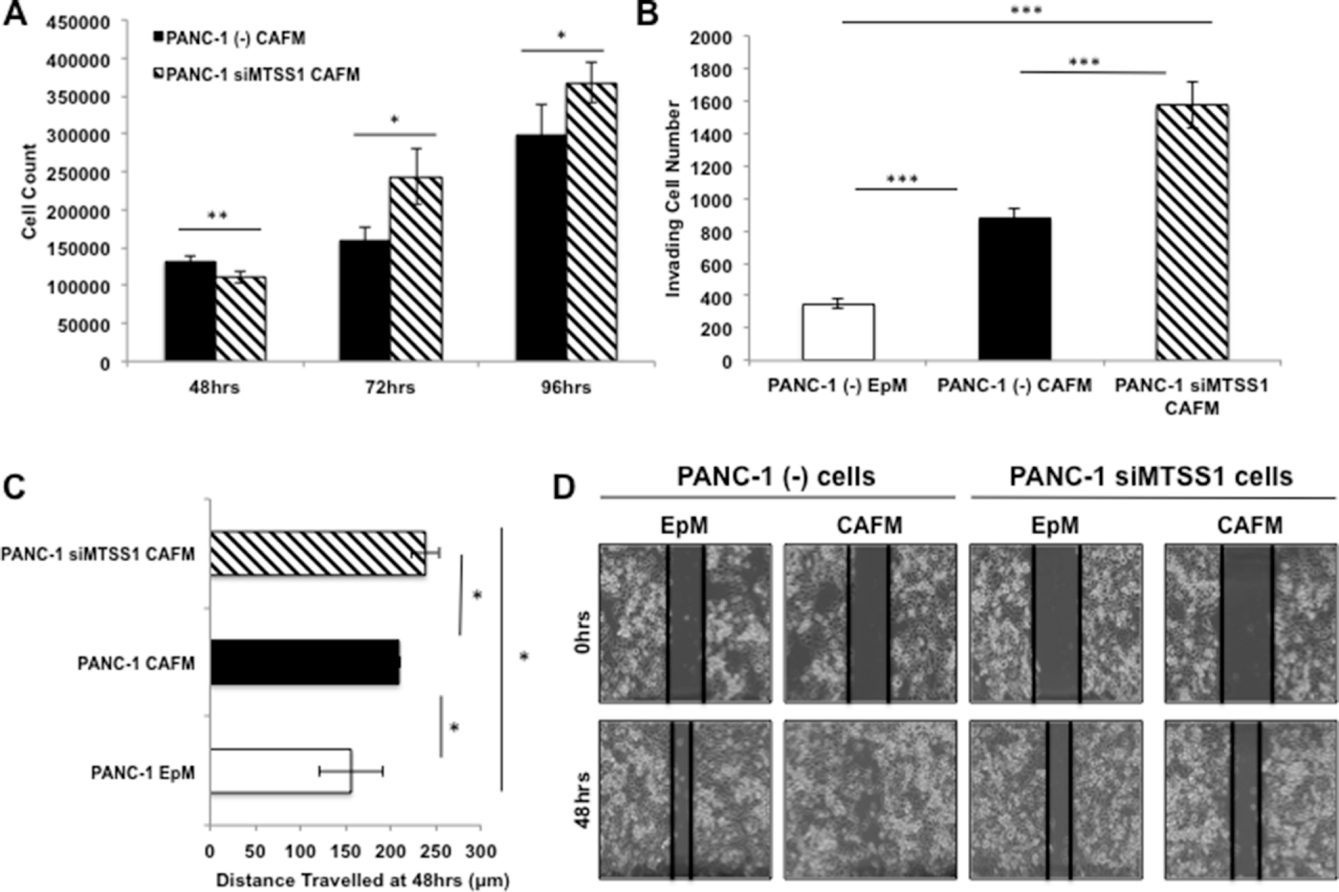
Increased metastatic phenotype seen with MTSS1 loss is augmented by CAF-conditioned media (**A**) Proliferation assay of PANC-1 (–) vs. PANC-1 siMTSS1 cells treated with CAFM for various timepoints. (**B**) PANC-1 cells were treated with either (–) control siRNA or siMTSS1 and then plated for a transwell migration assay in the presence of either EpM or CAFM. (**C**) PANC-1 (–) and siMTSS1 cells were treated in either EpM or CAFM and subjected to scratch assay conditions for 48 hours. (**D**) Representative images of PANC-1 (–) and PANC-1 siMTSS1 cells treated with either EpM or CAFM during scratch assay analysis. Images were taken at 10X magnification. *p-value < 0.05, **p-value < 0.001, ***p-value < 0.0001.

Having established that CAF-conditioned media affects the metastatic properties of PDAC cells, we next set out to establish if MTSS1 loss in PDAC cells treated with CAF-conditioned media enhances these effects. We performed functional assays to determine if MTSS1 knockdown and CAF-conditioned media treatment produced additive effects on cell proliferation, migration, and invasion. Proliferation assays revealed that siMTSS1 PANC-1 cells treated with CAFM have a significant increase in proliferation as compared to (–) control PANC-1 cells treated with the same CAFM (Figure 5A). Moreover, siMTSS1 PANC-1 cells treated with CAFM demonstrate a significant increase in invasion through a Matrigel-coated transwell membrane compared to (–) control PANC-1 cells in either EpM or CAFM, exhibiting a nearly 2-fold increase in cells that travelled through the membrane compared to (–) control cells treated with CAFM (Figure 5B, representative images, Supplementary Figure 9). Finally, we performed a scratch assay utilizing the same experimental setup. Our data show that CAFM-treated siMTSS1 PANC-1 cells migrate significantly farther as compared to EpM-treated PANC-1 cells and CAFM-treated PANC-1 cells (Figure 5C, representative images, Figure 5D).

In order to demonstrate that this increased invasion, migration, and proliferation was due to an additive effect of losing MTSS1 expression while also being treated with CAF-conditioned media, and not solely due to CAF-conditioned media increasing proliferation rates, we treated PANC-1 WT and MOE cells with either CAF-conditioned media or epithelial-conditioned media and performed a scratch invasion assay. PANC-1 WT cells treated with CAFM invaded approximately 350 μm farther than PANC-1 MOE cells treated with CAFM (Supplementary Figure 10). Similarly, PANC-1 WT cells treated with EpM were able to invade approximately 4-fold farther than PANC-1 MOE cells treated with EpM, which moved roughly as far as PANC-1 MOE cells treated with CAFM (Supplementary Figure 10). Additionally, we performed a scratch invasion assays on PANC-1 cells that were treated with either CAFM or serum-free CAFM. CAFs were plated in complete media, and then 24 hours post-plating, were placed in serum-free media, and then left to incubate for 72 hours before media harvest. Our data show that when PANC-1 cells are treated with either complete CAFM or serum-free CAFM, there is no difference in invasiveness (Supplementary Figure 11). These results show that reduction of MTSS1 expression in PDAC cells augments the increased cell proliferation, migration, and invasion seen in those cells treated with CAF-conditioned media.

### Increased MTSS1 expression in metastatic PDAC cells leads to increased survival *in vivo*

Our *in vitro* data shows that MTSS1 expression plays a key role in inhibiting cell migration and invasion of PDAC cells. We next set out to determine the effect of modulating MTSS1 expression in PDAC cells *in vivo*. Our initial analysis had identified MTSS1 as a gene whose expression predicted survival in patient dataset GSE32688. To provide additional validation of this finding, we examined another publically available PDAC patient dataset, GSE21501, to determine if MTSS1 expression correlated with patient outcome. We found that high MTSS1 expression was again revealed to be a significant predictor of better overall survival (HR: 0.7, *p* = .0051644; Supplementary Figure 12).

**Figure 6:**
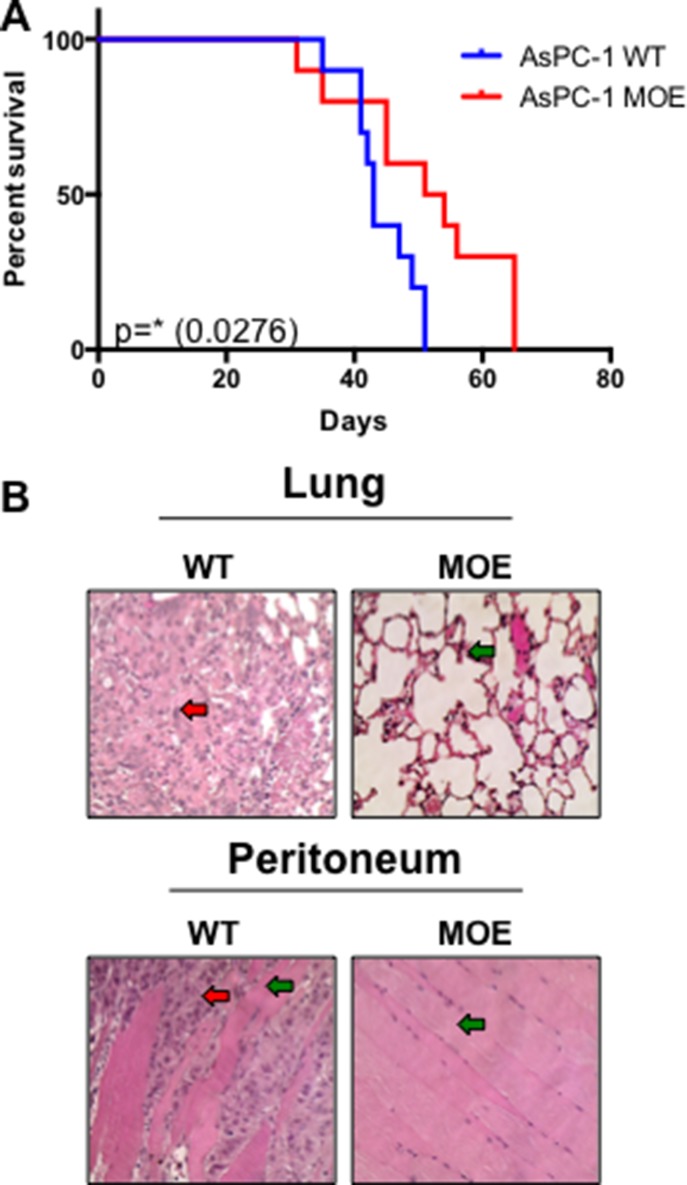
Increased MTSS1 expression leads to significantly prolonged survival *in vivo* (**A**) Kaplan-Meier survival curve of NOD-Scid mice injected intraperitoneally with either AsPC-1 WT or MOE cells. (**B**) H&E stained sections of tissues from AsPC-1 WT and MOE mice. H&E images were taken at 40x magnification (representative images from sacrifice at day 51). *p-value < 0.05.

Since this data strongly implicated MTSS1 in impacting the prognosis and survival of PDAC patients, we next explored the *in vivo* consequences of overexpressing MTSS1 in an intraperitoneal injection (IP) xenograft model of PDAC, which has been previously shown to closely mimic the metastatic progression of the clinical disease [36–38]. NOD/SCID/IL2γ null mice were intraperitoneally injected with either AsPC-1 WT cells or AsPC-1 MTSS1-overexpressing (MOE) cells that had been stably transduced with a MTSS1 retrovirus. Mice injected with AsPC-1 WT cells survived significantly shorter, on average 44.3 days, compared to mice injected with AsPC-1 MOE cells, which survived for 51.2 days on average (*p* = 0.0276; Figure 6A).

Additionally, histological examination of tissues taken from animals sacrificed at the same early time-point from both cohorts of mice revealed that mice injected with AsPC-1 WT cells had pronounced adenocarcinoma tumor cell presence in both the lung and the peritoneum (red arrow, representative images, Figure 6B and Supplementary Figure 13), organ commonly associated with PDAC metastasis [39], while mice injected with AsPC-1 MOE cells had normal lung and peritoneal tissue upon harvest (green arrow, representative images, Figure 6B and Supplementary Figure 13). Taken together, these data show that expression of MTSS1 plays a critical role in the suppression of cell invasion and migration *in vitro*; moreover, increasing MTSS1 expression in metastatic cells increases survival *in vivo*.

## DISCUSSION

In this study, we show that MTSS1 plays an important role in suppressing pancreatic cancer cell invasion and migration driven by the tumor microenvironment. We initially identified MTSS1 while searching for metastasis-related genes whose expression was linked to desmoplasia and poor prognosis in patients. In order to functionally determine the role MTSS1 plays in pancreatic cancer aggressiveness, we first showed that MTSS1 knockdown significantly increases PDAC cell migration and invasion. Conversely, we showed that MTSS1 overexpression leads to significantly decreased cell migration and invasion. Next, we demonstrated that CAF-conditioned media has the ability to increase the migration, invasion, and proliferation of PDAC cells, while also being able to decrease MTSS1 protein expression. Additionally, we showed that reduction of MTSS1 expression augments the increased metastatic potential seen in CAFM-treated PDAC cells. Finally, we demonstrated that increasing MTSS1 expression in metastatic cells increases survival in an *in vivo* model of metastatic pancreatic cancer.

PDAC is the 3rd leading cause of cancer-related deaths in the United States with a 5-year survival rate of 7% [1]. A major contributing factor to this poor prognosis is the fact that, currently, an astounding 53% of patients afflicted with PDAC are diagnosed at the metastatic stage [4]. This indicates that there is a critical need to better understand the molecular mechanisms that lead to metastasis. While data clearly show that inflammation in PDAC plays an important role as a driver of tumor cell dissemination that leads to metastasis [18], the molecular mechanisms responsible for this effect have yet to fully elucidated. Our data suggest that the desmoplastic stroma that is a hallmark of PDAC [40] suppresses MTSS1 activity, aiding tumor cell dissemination that can lead to metastasis.

The role in which the PDAC tumor microenvironment plays in PDAC progression has been enigmatic. There are multiple published studies indicating contradictory functions for the stroma, some suggesting it is pro-tumorigenic, others suggesting it is anti-tumorigenic. Some of these anti-tumorigenic findings have shown that depletion of the PDAC stroma via blocking hedgehog signaling can actually restrain metastatic progression [6, 41]. Different studies have yielded similar anti-tumorigenic findings where stromal depletion allowed for greater chemotherapy delivery and thus greater tumor cell death [42]. Conversely, the tumor stroma also has been studied in pro-tumorigenic fashion as well where researchers have found evidence of increased communication between the cells of the microenvironment and the tumor epithelium, resulting in its increased oncogenic potential [43, 44]. Needless to say, a better understanding of how the tumor stroma affects all stages of pancreatic tumorigenesis is critical for understanding how to better treat pancreatic cancer.

While a number of different studies have begun the process of characterizing the function of MTSS1 in a variety of cancer models, to our best knowledge, no functional characterization has been performed for MTSS1 in PDAC. In this study, we show that PDAC cell lines derived from previously characterized primary lesions display higher MTSS1 expression levels than PDAC cell lines derived from previously characterized metastatic lesions. This supports existing data that show that while *MTSS1* is expressed normally in early phases of tumorigenesis, it is lost in metastatic stages of disease [30–32, 45, 46]. We show that knockdown of MTSS1 leads to a more invasive and migratory phenotype in primary PDAC cells *in vitro*. Conversely, both transient and stable overexpression of MTSS1 in a metastatic PDAC cell line revert those cells to a more stationary, less migratory phenotype, and also lead to significantly increased survival *in vivo*.

There is conflicting data on whether MTSS1 expression is an indicator of good or poor prognosis in patients. Many studies have found that loss of MTSS1 is detrimental to patient survival and prognosis [32, 33, 47–50]; however, in both colorectal and hepatocellular carcinoma, data show that elevated MTSS1 is an indicator of poor prognosis [51, 52]. Our data show that in PDAC, high MTSS1 expression is a significant predictor of better overall survival in PDAC patients.

Interestingly, while our data show an increase in the metastatic potential of PDAC cells with decreased MTSS1 expression, intrinsically altering MTSS1 expression alone did not result in any difference in proliferation rates. This suggested that MTSS1 functions as a specific metastasis suppressor protein which regulates cell invasion and migration, as opposed to a canonical tumor suppressor protein which would also regulate cell proliferation and tumor growth [53]. However, when intrinsic loss of MTSS1 was coupled with exposure to CAF-conditioned media, increased proliferation of PDAC cells with decreased MTSS1 expression is observed. This increase in proliferation driven by CAFM conditions can be misconstrued as to being the sole reason why cells with decreased MTSS1 expression may migrate farther in a scratch assay. However, our data argue against this possibility since the results of the Matrigel transwell assay, one of the defining *in vitro* assay of metastasis [54, 55], mirrored the results of the scratch assays. In our studies, cells with decreased MTSS1 expression show in increase in cell invasion/migration. Thus, we believe our finding that loss of MTSS1 in the presence of CAF-conditioned media leads to increased invasion and migration in PDAC is based on increases in metastatic potential rather than proliferation rates. That being said, we cannot rule out the possibility that, when in concert with CAF-conditioned media, MTSS1 may play some role in increasing the proliferative ability of PDAC cells.

This additive phenomenon is important to note because a large majority of patients presenting with late stage disease have not only decreased *MTSS1* expression, but also the characteristically dense stromal microenvironment of PDAC. In another setting, perhaps MTSS1 loss would simply give cancer cells more of an advantage to disseminate from the primary tumor; however, in PDAC, where the CAF population is so massive, the loss of MTSS1 may not just aid in dissemination, but proliferation as well.

In this study, we show that CAF-conditioned media has the ability to increase the migration, invasion, and proliferation of PDAC cells. Metastasis suppressor proteins like MTSS1 could be critical for preventing the early dissemination of PDAC cells. Our data show that reduction of MTSS1 expression enhances the increased cell proliferation, migration, and invasion seen in PDAC cells treated with CAF-conditioned media. Our data suggest that the desmoplasia found during even the beginning stages of tumor formation eventually causes the downregulation of *MTSS1* in PDAC epithelial cells. Given that the stroma makes up the majority of cells in the tumor bulk in PDAC [56], it is possible that factors from the cancer-associated fibroblasts could lead to the selection of PDAC cells better able to migrate and invade.

We hypothesize that one of the key factors in generating the increased metastatic phenotype in pancreatic cancer cells is the major inflammatory response regulator, COX-2. It has previously been shown that co-culture of pancreatic cancer cells with fibroblasts induces COX-2 expression in the cancer cells [57]. One of the ways in which this COX-2 upregulation may lead to eventual loss of MTSS1 expression is through inflammation-mediated proteasome upregulation. Interleukin-6 (IL-6) is a known COX-2 dependent cytokine that exerts its effects through activation of oncogenic signaling pathways involved STAT3 [58, 59]. It has been shown that IL-6 upregulation can lead to increase in proteasome expression [60, 61]. This connection of COX-2 expression to proteasomal upregulation is key because it has been demonstrated that one of the ways MTSS1 is regulated is via proteasome ubiquitination [62]. If the inflammatory stromal microenvironment is responsible for upregulation of COX-2 in the pancreatic cancer cells, then it may also be responsible for the upregulation of the SCF^βTRCP^ proteasome, which leads to increased degradation and loss of MTSS1 expression.

Given the long latency between initial tumor cell development and metastasis in PDAC [63], it is likely multiple factors from the cancer-associated fibroblasts could influence MTSS1 levels in PDAC cells over time. The exact mechanisms through which the stroma may exert its effect on MTSS1 are still unknown. Studies show that multiple microRNAs are capable of regulating MTSS1 expression [64–67]. Moreover, as previously mentioned, ubiquitination-driven destruction and DNA methylation have also been found to play a role in the downregulation of MTSS1 [62, 68, 69]. Future studies are needed to elucidate the relationship stromal-cell released factors and MTSS1 expression in PDAC.

In summary, increased desmoplasia in PDAC tumors corresponds to lower levels of the metastasis suppressor protein, MTSS1. Knockdown of MTSS1 expression leads to increased PDAC cell invasion and migration, whereas overexpression of MTSS1 in PDAC cell lines halts migration and invasion significantly. MTSS1 overexpression in PDAC cells also leads to significantly increased survival *in vivo*. Increased migration and invasion of PDAC cells is also seen when these cells are treated with CAF-conditioned media. Moreover, there is an additive effect seen when cells are both MTSS1-deficient and treated with CAF media. These findings indicate a cell-extrinsic mechanism for the regulation of MTSS1 in PDAC and help elucidate the process through which PDAC cells enhance properties that promote tumor cell dissemination in this deadly disease.

## MATERIALS AND METHODS

### Cell culture

Patient-derived, cancer-associated fibroblast (CAF) line UH1301-63 (S63) was obtained from Melissa L. Fishel, Ph.D. (Department of Pediatrics, IU School of Medicine) and prepared for study as previously described [70]. PANC-1, MIA-PaCa, L3.6, S63, and BxPC3 cells were cultured in DMEM supplemented with 10% FBS, 100 U/mL of penicillin, and 100 μg/mL of streptomycin with 5% CO_2_ at 37°C. AsPC-1 and Hs-766T cells were cultured in RPMI supplemented with 10% FBS, 100 U/mL of penicillin, and 100 μg/mL of streptomycin with 5% CO_2_ at 37°C. Cells were trypsinized and put in fresh complete media at approximately 90% confluence.

### Cell authentication and sequencing

Cell line authentication of both fibroblast and epithelial cell lines was completed by Genetica DNA Laboratories. All epithelial cell lines were determined to be a 97%–100% match to the appropriate cell line in both the ATCC and DSMZ database. The L3.6 cell line, which was graciously shared by Dr. Timothy Donahue from UCLA, is an epithelial patient-derived cell line [71]. The cancer-associated fibroblast cell line used in these studies was determined not to have a match in either the ATCC or DSMZ cell line database. These cells were also determined to be non-tumorigenic in mice and did not harbor a mutation for *K-RAS* [70], suggesting no cell contamination with epithelial cells.

### Mouse/human array comparison analysis

320 differentially expressed genes (+/– 2-fold change) in our mouse model of PDAC based on the presence or absence of COX-2 [24] (GSE38988), were compared to a list of genes identified in an Affymetrix analysis of human patient samples indicative of poor prognosis [28] (GSE32688), in order to identify 17 candidate genes that were linked with inflammation and poor prognosis.

### Patient dataset analysis

The prognostic value of MTSS1 in PDAC patients was analyzed in publically available microarray dataset, GSE21501 [72], using PROGgene gene expression based survival analysis web application [73].

### Retrovirus production and transduction

293-T cells at 60% confluence were transfected with vector control plasmid (5 μg), WT MTSS1 plasmid (5 μg), or MTSS1 S322A plasmid (5 μg). VSVG (3 μg) and GAG (2 μg) plasmids along with Lipofectamine 2000 (10 μg) (Qiagen) were added to the plasmids of interest in order to gain properly packaged retrovirus that was collected 48 hours later. Harvested retroviral media was then added to 70% confluent AsPC-1 cells supplemented with polybrene (1:1000 dilution). Treated cells were then centrifuged for at 2500rpm for one hour for two days post-transduction. Verification of MTSS1 expression was accomplished via qRT-PCR and western blot analysis.

### Murine *in vivo* survival study

1 × 10^6^ WT and retrovirally transduced AsPC-1 cells to overexpress MTSS1 were diluted in 500 uL serum-free RPMI media and IP injected into male NOD/SCID/IL2γ null mice using 25-gauge syringe. Tumors were allowed to develop until death or necessary sacrifice was warranted according to protocol. The mice were divided into 2 cohorts, 10 mice per cohort: (1) AsPC-1 WT control and (2) AsPC-1 MTSS1-overexpression (MOE). Mice were monitored daily and, upon morbidity, were sacrificed. Time of death was determined from the day of the cellular injection. Statistical analysis of *in vivo* data was completed using GraphPad Prism Version 6.0 software, specifically the Log-rank (Mantel-Cox) test.

### Immunohistochemistry

Immunohistochemical analysis was conducted on formalin-fixed, paraffin-embedded tissue. Tissue was harvested from freshly sacrificed mice and left in 10% formalin overnight at room temperature. Tissue was then rinsed with deionized water for approximately 3 minutes and placed in 70% ethanol until ready for staining. Staining was completed by the University of Notre Dame Integrated Imaging Facility Histology Core, and all slides were reviewed by a board certified pathologist before analysis.

### siRNA transfection

Cells were plated at a density of 200,000 epithelial or 100,000 fibroblast cells/well in 6-well plates (Corning) and transfected with 100 nM siRNA 24 hours post-plating using HiPerfect transfection reagent (Qiagen). The following siRNAs were used: Murine MTSS1 (Qiagen GS211401) and Human MTSS1 (Qiagen GS9788). The siRNA sequences that were used are as follows: Hs_MTSS1_5 (SI02757755) CCGACG GATGTTCCAAGCCAA, Hs_MTSS1_6 (SI04438392) TCCCGTCATCTCAGATCCCTA, Hs_MTSS1_4 (SI00 107954) CCGTATGGTCATTGTTCTATA, Hs_MTSS1_3 (SI00107947) ACAGGTGATTCTGGACTTGAA. Murine siRNA was used initially before human siRNA became available. Results were the same regardless of siRNA used; however, all figures represent assays completed using the human MTSS1 siRNA. Homology of MTSS1 gene between mouse and human is 96% [69], and sufficient knockdown (∼30%–50%) was achieved using both methods. 48 hours post-transfection, cells were either harvested for validation or replated for additional analysis.

### RNA collection and qRT-PCR

RNA from cells was harvested via Trizol according to standard protocol, resuspended in RNAase free water and analyzed for purity by 260/280 absorbance via nanodrop. qRT-PCR was conducted on the BioRad-CFX-Connect cycler using Qiagen OneStep RT-PCR kit (Qiagen 210210) and the following primers: Human GAPDH (Qiagen PPH00150F) and Human MTSS1 (Qiagen PPH10073B).

### Western blot

Cell lysates were lysed in radioimmunoprecipitation (RIPA) buffer supplemented with a protease inhibitor cocktail tablet (Roche 11836153001). Concentration was verified by BCA analysis (BioRad) and subjected to gel electrophoresis (BioRad Mini Protean TGX Gel 400091313) and transferred to a nitrocellulose membrane. The membranes were blocked in 5% dry milk in TBS-T. Primary antibodies diluted in 1% dry milk and TBS-T used includes: MTSS1 (Cell Signaling 4386S), COX-2 (Cell Signaling 12282S), and β-Actin (Cell Signaling 4970L) at 1:1000. Secondary antibodies used include: Anti-Rabbit (Cell Signaling 7074S). Protein levels were detected by enhanced chemiluminescence (ECL) (Thermo Scientific 32106) and quantitated with Image Lab software (Bio-Rad). Densitometry analysis was completed using ImageJ software.

### Conditioned media collection

Media from cancer-associated fibroblasts or PANC-1 cells was collected after 3–4 days at 80–90% confluence and centrifuged at 2,500 RPM for 5 minutes in order to pellet any debris. Media was stored at 4°C until needed. For serum-free CAFM studies, CAF cells were plated in complete DMEM for 24 hours, then washed with PBS and placed in serum-free DMEM for 72 hours. Media was collected and centrifuged at 2,500 RPM for 5 minutes in order to pellet any debris.

### Transwell assays

40,000 epithelial cells (25,000 cancer-associated fibroblasts) were plated on a 24-well transwell polycarbonate membrane with 8.0 μm pore size (Corning 3422). Wells used for migratory studies were coated with 50 μL of 3 mg/mL Matrigel (Corning 354230) and kept at 37°C for 24 hours before plating to ensure solidification. Cells plated in the upper chamber, unless treated with conditioned media, were placed in 150 μL of the appropriate serum-free media, whereas the bottom chamber contained 700 μL of the appropriate serum-containing media to act as the chemoattractant in the study. Once the cells were plated, the assay was run for 48 hours before cells were briefly and gently rinsed with ddH_2_O and then fixed for 20 minutes with 10% formalin (Azer Scientific). After fixation, the cells were placed in hematoxylin (Sigma) for 2 minutes, quickly rinsed in ddH_2_O, quickly dipped in 1% acid alcohol (Sigma), and rinsed a final time in ddH_2_O. Membranes were then imaged and individual cells were counted manually using an AMG EVOS XL Core Cell Imaging System microscope (AMEX1000).

### Scratch assays

Cells were plated in 6-well plates (Corning) and allowed to reach 90% confluence. Once at the appropriate confluence, media was removed from the wells and scratches were made down the center of the well using a P10 pipette tip. Cells were then washed twice with 1X PBS (Sigma). Once the PBS was removed, 2 mL of serum-free, serum-containing, or conditioned media was added to the appropriate wells. Images were taken at 0, 12, 24, and 48 hours post-scratch using the AMG EVOS FL Cell Imaging System microscope (AMEX4300). Analysis was completed in triplicate using ImageJ.

### Statistical analysis

Experiments were performed with a minimum of three biological replicates. Data are presented as the mean ± standard deviation. Statistical significance was calculated via Microsoft Excel using a Student *t* test or ANOVA as appropriate. *Denotes *p*-value < 0.05 **denotes *p*-value < 0.01 ***denotes *p*-value < 0.0001

## SUPPLEMENTARY MATERIALS FIGURES AND TABLES



## References

[1] Stathis A, Moore MJ (2010). Advanced pancreatic carcinoma: current treatment and future challenges. Nat Rev Clin Oncol.

[2] Siegel RL, Miller KD, Jemal A (2016). Cancer statistics, 2016. CA Cancer J Clin.

[3] Rahib L, Smith BD, Aizenberg R, Rosenzweig AB, Fleshman JM, Matrisian LM (2014). Projecting cancer incidence and deaths to 2030: the unexpected burden of thyroid, liver, and pancreas cancers in the United States. Cancer Res.

[4] Steeg PS (2016). Targeting metastasis. Nat Rev Cancer.

[5] Oettle H, Post S, Neuhaus P, Gellert K, Langrehr J, Ridwelski K, Schramm H, Fahlke J, Zuelke C, Burkart C, Gutberlet K, Kettner E, Schmalenberg H (2007). Adjuvant chemotherapy with gemcitabine vs observation in patients undergoing curative-intent resection of pancreatic cancer: a randomized controlled trial. JAMA.

[6] Rhim AD, Oberstein PE, Thomas DH, Mirek ET, Palermo CF, Sastra SA, Dekleva EN, Saunders T, Becerra CP, Tattersall IW, Westphalen CB, Kitajewski J, Fernandez-Barrena MG (2014). Stromal elements act to restrain, rather than support, pancreatic ductal adenocarcinoma. Cancer Cell.

[7] Lowenfels AB, Maisonneuve P, Cavallini G, Ammann RW, Lankisch PG, Andersen JR, Dimagno EP, Andren-Sandberg A, Domellof L (1993). Pancreatitis and the risk of pancreatic cancer. International Pancreatitis Study Group. N Engl J Med.

[8] Malka D, Hammel P, Maire F, Rufat P, Madeira I, Pessione F, Levy P, Ruszniewski P (2002). Risk of pancreatic adenocarcinoma in chronic pancreatitis. Gut.

[9] Raimondi S, Lowenfels AB, Morselli-Labate AM, Maisonneuve P, Pezzilli R (2010). Pancreatic cancer in chronic pancreatitis; aetiology, incidence, and early detection. Best Pract Res Clin Gastroenterol.

[10] Loncle C, Molejon MI, Lac S, Tellechea JI, Lomberk G, Gramatica L, MF Fernandez Zapico, Dusetti N, Urrutia R, Iovanna JL (2016). The pancreatitis-associated protein VMP1, a key regulator of inducible autophagy, promotes Kras(G12D)-mediated pancreatic cancer initiation. Cell Death Dis.

[11] Loncle C, Bonjoch L, Folch-Puy E, Lopez-Millan MB, Lac S, Molejon MI, Chuluyan E, Cordelier P, Dubus P, Lomberk G, Urrutia R, Closa D, Iovanna JL (2015). IL17 Functions through the Novel REG3beta-JAK2-STAT3 Inflammatory Pathway to Promote the Transition from Chronic Pancreatitis to Pancreatic Cancer. Cancer Res.

[12] Mohammed A, Janakiram NB, Madka V, Brewer M, Ritchie RL, Lightfoot S, Kumar G, Sadeghi M, Patlolla JM, Yamada HY, Cruz-Monserrate Z, May R, Houchen CW (2015). Targeting pancreatitis blocks tumor-initiating stem cells and pancreatic cancer progression. Oncotarget.

[13] Hwang RF, Moore T, Arumugam T, Ramachandran V, Amos KD, Rivera A, Ji B, Evans DB, Logsdon CD (2008). Cancer-associated stromal fibroblasts promote pancreatic tumor progression. Cancer Res.

[14] Olive KP, Jacobetz MA, Davidson CJ, Gopinathan A, McIntyre D, Honess D, Madhu B, Goldgraben MA, Caldwell ME, Allard D, Frese KK, Denicola G, Feig C (2009). Inhibition of Hedgehog signaling enhances delivery of chemotherapy in a mouse model of pancreatic cancer. Science.

[15] Kawase T, Yasui Y, Nishina S, Hara Y, Yanatori I, Tomiyama Y, Nakashima Y, Yoshida K, Kishi F, Nakamura M, Hino K (2015). Fibroblast activation protein-alpha-expressing fibroblasts promote the progression of pancreatic ductal adenocarcinoma. BMC Gastroenterol.

[16] Kalluri R, Zeisberg M (2006). Fibroblasts in cancer. Nature Rev Cancer.

[17] Vonlaufen A, Phillips PA, Xu Z, Goldstein D, Pirola RC, Wilson JS, Apte MV (2008). Pancreatic stellate cells and pancreatic cancer cells: an unholy alliance. Cancer Res.

[18] Rhim AD, Mirek ET, Aiello NM, Maitra A, Bailey JM, McAllister F, Reichert M, Beatty GL, Rustgi AK, Vonderheide RH, Leach SD, Stanger BZ (2012). EMT and dissemination precede pancreatic tumor formation. Cell.

[19] Heeg S, Das KK, Reichert M, Bakir B, Takano S, Caspers J, Aiello NM, Wu K, Neesse A, Maitra A, Iacobuzio-Donahue CA, Hicks P, Rustgi AK (2016). ETS-Transcription Factor ETV1 Regulates Stromal Expansion and Metastasis in Pancreatic Cancer. Gastroenterology.

[20] Hill R, Calvopina JH, Kim C, Wang Y, Dawson DW, Donahue TR, Dry S, Wu H (2010). PTEN loss accelerates KrasG12D-induced pancreatic cancer development. Cancer Res.

[21] Hla T, Bishop-Bailey D, Liu CH, Schaefers HJ, Trifan OC (1999). Cyclooxygenase-1 and -2 isoenzymes. Int J Biochem Cell Biol.

[22] Schlosser W, Schlosser S, Ramadani M, Gansauge F, Gansauge S, Beger HG (2002). Cyclooxygenase-2 is overexpressed in chronic pancreatitis. Pancreas.

[23] Tucker ON, Dannenberg AJ, Yang EK, Zhang F, Teng L, Daly JM, Soslow RA, Masferrer JL, Woerner BM, Koki AT, Fahey TJ (1999). Cyclooxygenase-2 expression is up-regulated in human pancreatic cancer. Cancer Res.

[24] Hill R, Li Y, Tran LM, Dry S, Calvopina JH, Garcia A, Kim C, Wang Y, Donahue TR, Herschman HR, Wu H (2012). Cell intrinsic role of COX-2 in pancreatic cancer development. Mol Cancer Ther.

[25] Kundu N, Yang Q, Dorsey R, Fulton AM (2001). Increased cyclooxygenase-2 (cox-2) expression and activity in a murine model of metastatic breast cancer. Int J Cancer.

[26] Cheng J, Fan XM (2013). Role of cyclooxygenase-2 in gastric cancer development and progression. World J Gastroenterol.

[27] Chen WS, Wei SJ, Liu JM, Hsiao M, Kou-Lin J, Yang WK (2001). Tumor invasiveness and liver metastasis of colon cancer cells correlated with cyclooxygenase-2 (COX-2) expression and inhibited by a COX-2-selective inhibitor, etodolac. Int J Cancer.

[28] Donahue TR, Tran LM, Hill R, Li Y, Kovochich A, Calvopina JH, Patel SG, Wu N, Hindoyan A, Farrell JJ, Li X, Dawson DW, Wu H (2012). Integrative survival-based molecular profiling of human pancreatic cancer. Clin Cancer Res.

[29] Dawson JC, Timpson P, Kalna G, Machesky LM (2012). Mtss1 regulates epidermal growth factor signaling in head and neck squamous carcinoma cells. Oncogene.

[30] Lee YG, Macoska JA, Korenchuk S, Pienta KJ (2002). MIM, a potential metastasis suppressor gene in bladder cancer. Neoplasia.

[31] Loberg RD, Neeley CK, Adam-Day LL, Fridman Y, LN St John, Nixdorf S, Jackson P, Kalikin LM, Pienta KJ (2005). Differential expression analysis of MIM (MTSS1) splice variants and a functional role of MIM in prostate cancer cell biology. Int J Oncol.

[32] Parr C, Jiang WG (2009). Metastasis suppressor 1 (MTSS1) demonstrates prognostic value and anti-metastatic properties in breast cancer. Eur J Cancer.

[33] Liu K, Wang G, Ding H, Chen Y, Yu G, Wang J (2010). Downregulation of metastasis suppressor 1(MTSS1) is associated with nodal metastasis and poor outcome in Chinese patients with gastric cancer. BMC Cancer.

[34] Deer EL, Gonzalez-Hernandez J, Coursen JD, Shea JE, Ngatia J, Scaife CL, Firpo MA, Mulvihill SJ (2010). Phenotype and genotype of pancreatic cancer cell lines. Pancreas.

[35] Bruns CJ, Harbison MT, Kuniyasu H, Eue I, In Fidler IJ (1999). vivo selection and characterization of metastatic variants from human pancreatic adenocarcinoma by using orthotopic implantation in nude mice. Neoplasia.

[36] Awasthi N, Scire E, Monahan S, Grojean M, Zhang E, Schwarz MA, Schwarz RE (2016). Augmentation of response to nab-paclitaxel by inhibition of insulin-like growth factor (IGF) signaling in preclinical pancreatic cancer models. Oncotarget.

[37] Awasthi N, Zhang C, Schwarz AM, Hinz S, Wang C, Williams NS, Schwarz MA, Schwarz RE (2013). Comparative benefits of Nab-paclitaxel over gemcitabine or polysorbate-based docetaxel in experimental pancreatic cancer. Carcinogenesis.

[38] Ostapoff KT, Awasthi N, Cenik BK, Hinz S, Dredge K, Schwarz RE, Brekken RA (2013). PG545, an angiogenesis and heparanase inhibitor, reduces primary tumor growth and metastasis in experimental pancreatic cancer. Mol Cancer Ther.

[39] Cannistra M, Ruggiero M, Zullo A, Serafini S, Grande R, Nardo B (2015). Metastases of pancreatic adenocarcinoma: A systematic review of literature and a new functional concept. Int J Surg.

[40] Korc M (2007). Pancreatic cancer-associated stroma production. Am J Surg.

[41] Lee JJ, Perera RM, Wang H, Wu DC, Liu XS, Han S, Fitamant J, Jones PD, Ghanta KS, Kawano S, Nagle JM, Deshpande V, Boucher Y (2014). Stromal response to Hedgehog signaling restrains pancreatic cancer progression. PNAS.

[42] Provenzano PP, Cuevas C, Chang AE, Goel VK, Von Hoff DD, Hingorani SR (2012). Enzymatic targeting of the stroma ablates physical barriers to treatment of pancreatic ductal adenocarcinoma. Cancer Cell.

[43] Iacobuzio-Donahue CA, Ryu B, Hruban RH, Kern SE (2002). Exploring the host desmoplastic response to pancreatic carcinoma: gene expression of stromal and neoplastic cells at the site of primary invasion. Am J Pathol.

[44] Ricci F, Kern SE, Hruban RH, Iacobuzio-Donahue CA (2005). Stromal responses to carcinomas of the pancreas: juxtatumoral gene expression conforms to the infiltrating pattern and not the biologic subtype. Cancer Biol Ther.

[45] Callahan CA, Ofstad T, Horng L, Wang JK, Zhen HH, Coulombe PA, Oro AE (2004). MIM/BEG4, a Sonic hedgehog-responsive gene that potentiates Gli-dependent transcription. Genes Dev.

[46] Zhang K, Jiao X, Liu X, Zhang B, Wang J, Wang Q, Tao Y, Zhang D (2010). Knockdown of snail sensitizes pancreatic cancer cells to chemotherapeutic agents and irradiation. Int J Mol Sci.

[47] Shi W, Hasimu G, Wang Y, Li N, Chen M, Zhang H (2015). MTSS1 is an independent prognostic biomarker for survival in intrahepatic cholangiocarcinoma patients. Am J Transl Res.

[48] Zhou L, Li J, Shao QQ, Guo JC, Liang ZY, Zhou WX, Zhang TP, You L, Zhao YP (2016). Expression and Significances of MTSS1 in Pancreatic Cancer. Pathol Oncol Res.

[49] Zhang S, Qi Q (2015). MTSS1 suppresses cell migration and invasion by targeting CTTN in glioblastoma. J Neurooncol.

[50] Wang F, Liu Y, Zhang H (2013). Loss of MTSS1 expression is an independent prognostic factor for Hilar cholangiocarcinoma. Pathol Oncol Res.

[51] Huang XY, Huang ZL, Xu B, Chen Z, Re TJ, Zheng Q, Tang ZY, Huang XY (2016). Elevated MTSS1 expression associated with metastasis and poor prognosis of residual hepatitis B-related hepatocellular carcinoma. J Exp Clin Cancer Res.

[52] Wang D, Xu MR, Wang T, Li T, Zhu J (2011). MTSS1 overexpression correlates with poor prognosis in colorectal cancer. J Gastrointest Surg.

[53] Hurst DR, Welch DR (2011). Metastasis suppressor genes at the interface between the environment and tumor cell growth. Int Rev Cell Mol Biol.

[54] Albini A, Iwamoto Y, Kleinman HK, Martin GR, Aaronson SA, Kozlowski JM, Mcewan RN (1987). A Rapid Invitro Assay for Quantitating the Invasive Potential of Tumor-Cells. Cancer Res.

[55] Kramer N, Walzl A, Unger C, Rosner M, Krupitza G, Hengstschlager M, In Dolznig H (2013). vitro cell migration and invasion assays. Mutat Res-Rev Mutat.

[56] Kleeff J, Beckhove P, Esposito I, Herzig S, Huber PE, Lohr JM, Friess H (2007). Pancreatic cancer microenvironment. Int J cancer.

[57] Matsubayashi H, Infante JR, Winter J, Klein AP, Schulick R, Hruban R, Visvanathan K, Goggins M (2007). Tumor COX-2 expression and prognosis of patients with resectable pancreatic cancer. Cancer Biol Ther.

[58] Kabir TD, Leigh RJ, Tasena H, Mellone M, Coletta RD, Parkinson EK, Prime SS, Thomas GJ, Paterson IC, Zhou D, McCall J, Speight PM, Lambert DW (2016). A miR-335/COX-2/PTEN axis regulates the secretory phenotype of senescent cancer-associated fibroblasts. Aging.

[59] Dalwadi H, Krysan K, Heuze-Vourc'h N, Dohadwala M, Elashoff D, Sharma S, Cacalano N, Lichtenstein A, Dubinett S (2005). Cyclooxygenase-2-dependent activation of signal transducer and activator of transcription 3 by interleukin-6 in non-small cell lung cancer. Clin Cancer Res.

[60] Fujita J, Tsujinaka T, Yano M, Ebisui C, Saito H, Katsume A, Akamatsu K, Ohsugi Y, Shiozaki H, Monden M (1996). Anti-interleukin-6 receptor antibody prevents muscle atrophy in colon-26 adenocarcinoma-bearing mice with modulation of lysosomal and ATP-ubiquitin-dependent proteolytic pathways. Int J Cancer.

[61] Narsale AA, Carson JA (2014). Role of interleukin-6 in cachexia: therapeutic implications. Curr Opin Support Palliat Care.

[62] Zhong J, Shaik S, Wan L, Tron AE, Wang Z, Sun L, Inuzuka H, Wei W (2013). SCF beta-TRCP targets MTSS1 for ubiquitination-mediated destruction to regulate cancer cell proliferation and migration. Oncotarget.

[63] Yachida S, Jones S, Bozic I, Antal T, Leary R, Fu B, Kamiyama M, Hruban RH, Eshleman JR, Nowak MA, Velculescu VE, Kinzler KW, Vogelstein B (2010). Distant metastasis occurs late during the genetic evolution of pancreatic cancer. Nature.

[64] Kedmi M, Ben-Chetrit N, Korner C, Mancini M, Ben-Moshe NB, Lauriola M, Lavi S, Biagioni F, Carvalho S, Cohen-Dvashi H, Schmitt F, Wiemann S, Blandino G (2015). EGF induces microRNAs that target suppressors of cell migration: miR-15b targets MTSS1 in breast cancer. Sci Signal.

[65] Wu W, Wang Z, Yang P, Yang J, Liang J, Chen Y, Wang H, Wei G, Ye S, Zhou Y (2014). MicroRNA-135b regulates metastasis suppressor 1 expression and promotes migration and invasion in colorectal cancer. Mol Cell Biochem.

[66] Jahid S, Sun J, Edwards RA, Dizon D, Panarelli NC, Milsom JW, Sikandar SS, Gumus ZH, Lipkin SM (2012). miR-23a promotes the transition from indolent to invasive colorectal cancer. Cancer Discov.

[67] Wang J, Li J, Shen J, Wang C, Yang L, Zhang X (2012). MicroRNA-182 downregulates metastasis suppressor 1 and contributes to metastasis of hepatocellular carcinoma. BMC Cancer.

[68] Fan H, Chen L, Zhang F, Quan Y, Su X, Qiu X, Zhao Z, Kong KL, Dong S, Song Y, Chan TH, Guan XY (2012). MTSS1, a novel target of DNA methyltransferase 3B, functions as a tumor suppressor in hepatocellular carcinoma. Oncogene.

[69] Utikal J, Gratchev A, Muller-Molinet I, Oerther S, Kzhyshkowska J, Arens N, Grobholz R, Kannookadan S, Goerdt S (2006). The expression of metastasis suppressor MIM/MTSS1 is regulated by DNA methylation. Int J Cancer.

[70] Richards K, Zeleniak A, Fishel M, Wu J, Littlepage L, Hill R (2016). Cancer-Associated Fibroblast Exosomes Regulate Survival and Proliferation of Pancreatic Cancer Cells. Oncogene.

[71] Arensman MD, Kovochich AN, Kulikauskas RM, Lay AR, Yang PT, Li X, Donahue T, Major MB, Moon RT, Chien AJ, Dawson DW (2014). WNT7B mediates autocrine Wnt/beta-catenin signaling and anchorage-independent growth in pancreatic adenocarcinoma. Oncogene.

[72] Stratford JK, Bentrem DJ, Anderson JM, Fan C, Volmar KA, Marron JS, Routh ED, Caskey LS, Samuel JC, LB Der CJ Thorne, Calvo BF, Kim HJ (2010). A six-gene signature predicts survival of patients with localized pancreatic ductal adenocarcinoma. PLoS Med.

[73] Goswami CP, Nakshatri H (2013). PROGgene: gene expression based survival analysis web application for multiple cancers. J Clin Bioinforma.

