# Supplementary material for: Loss of MTSS1 results in increased metastatic potential in pancreatic cancer
Source: Oncotarget. 2017 Jan 27;8(10):16473–87. doi: 10.18632/oncotarget.14869 (PMC5369978; doi:10.18632/oncotarget.14869)
Supplement: Supplementary file 1 [file oncotarget-08-16473-s001.pdf]

Loss of MTSS1 results in increased metastatic potential in pancreatic cancer

Supplementary Materials

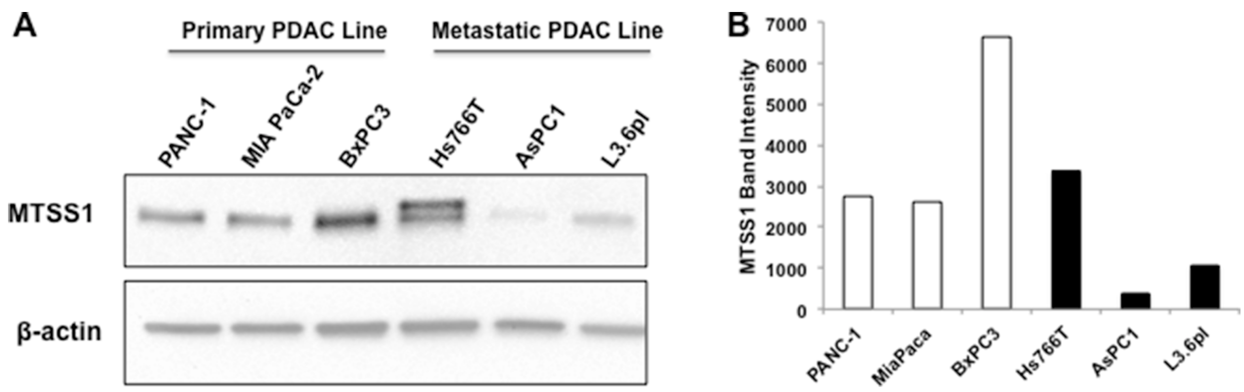

Supplementary Figure 1: MTSS1 expression is lower in PDAC cell lines derived from primary tumor sites. (**A**) Western blot analysis of MTSS1 expression in a panel of PDAC cell lines that were either derived from a primary PDAC cancer site or a metastatic PDAC cancer site. (**B**) Densitometry analysis of PDAC panel western blot for MTSS1 expression levels.

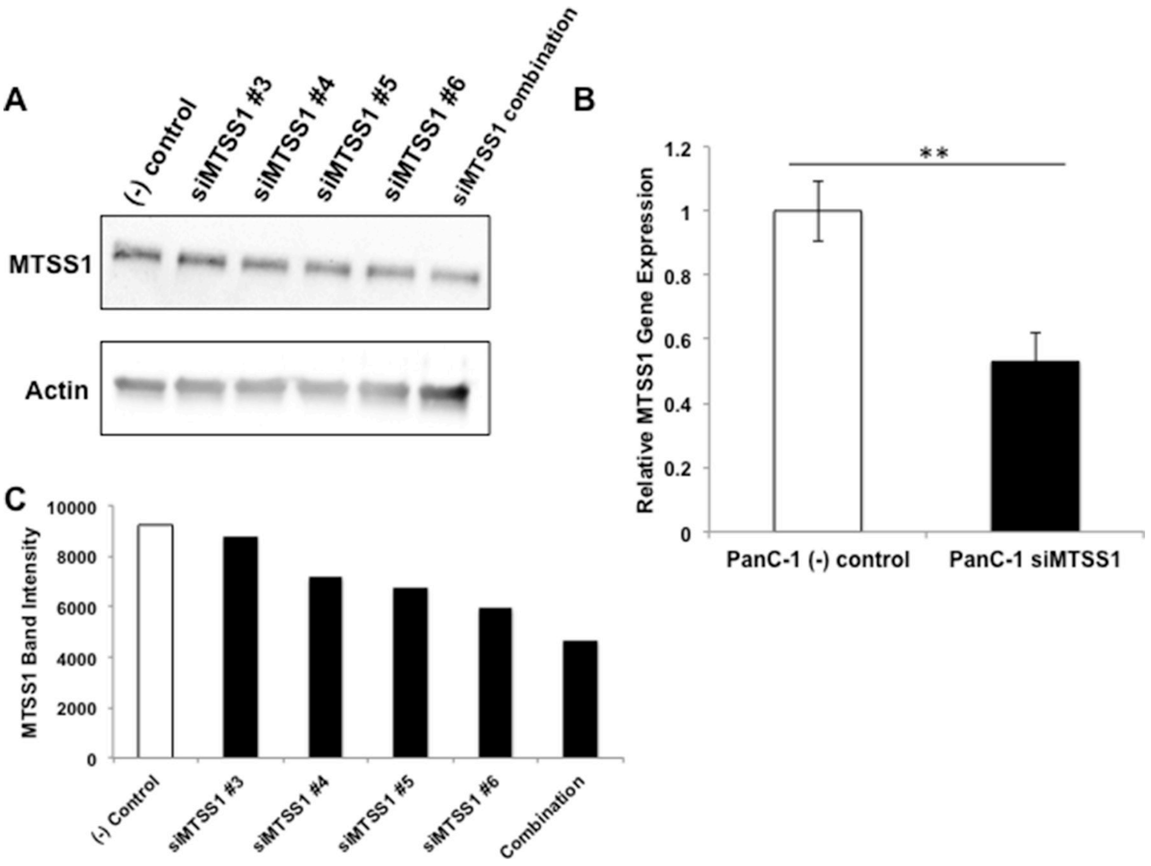

Supplementary Figure 2: Quantification of MTSS1 knockdown using MTSS1 siRNA combination. (**A**) Western blot analysis of PANC-1 cells treated with a panel or combination of MTSS1 siRNA. Greatest knockdown was achieved when using the siMTSS1 combination treatment (~50%), and thus this was the siRNA method used for all transfections. (**B**) RT-PCR analysis of siRNA knockdown of MTSS1 in PANC-1 cells using siMTSS1 combination treatment. (**C**) Quantitative analysis of MTSS1 expression during western blot knockdown validation seen in Supplementary Figure 2A. ***p*-value < 0.001.

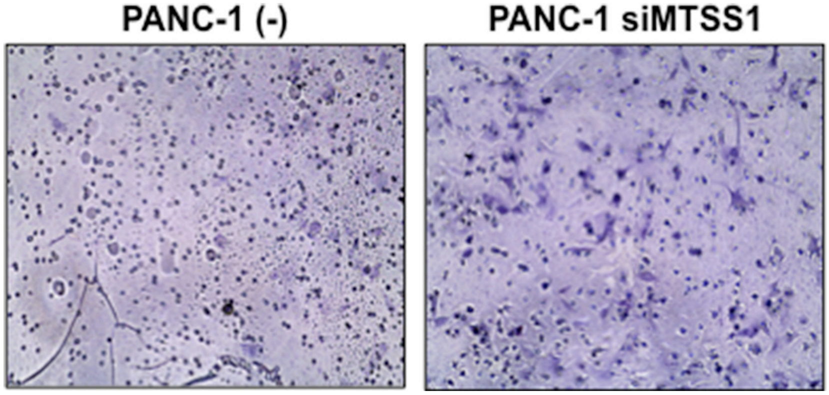

Supplementary Figure 3: Loss of MTSS1 leads to more cell invasion in a transwell assay. Representative images of Matrigel-coated transwell migration assay between PANC-1 (–) cells and PANC-1 siMTSS1 cells. Images were taken at 20× magnification.

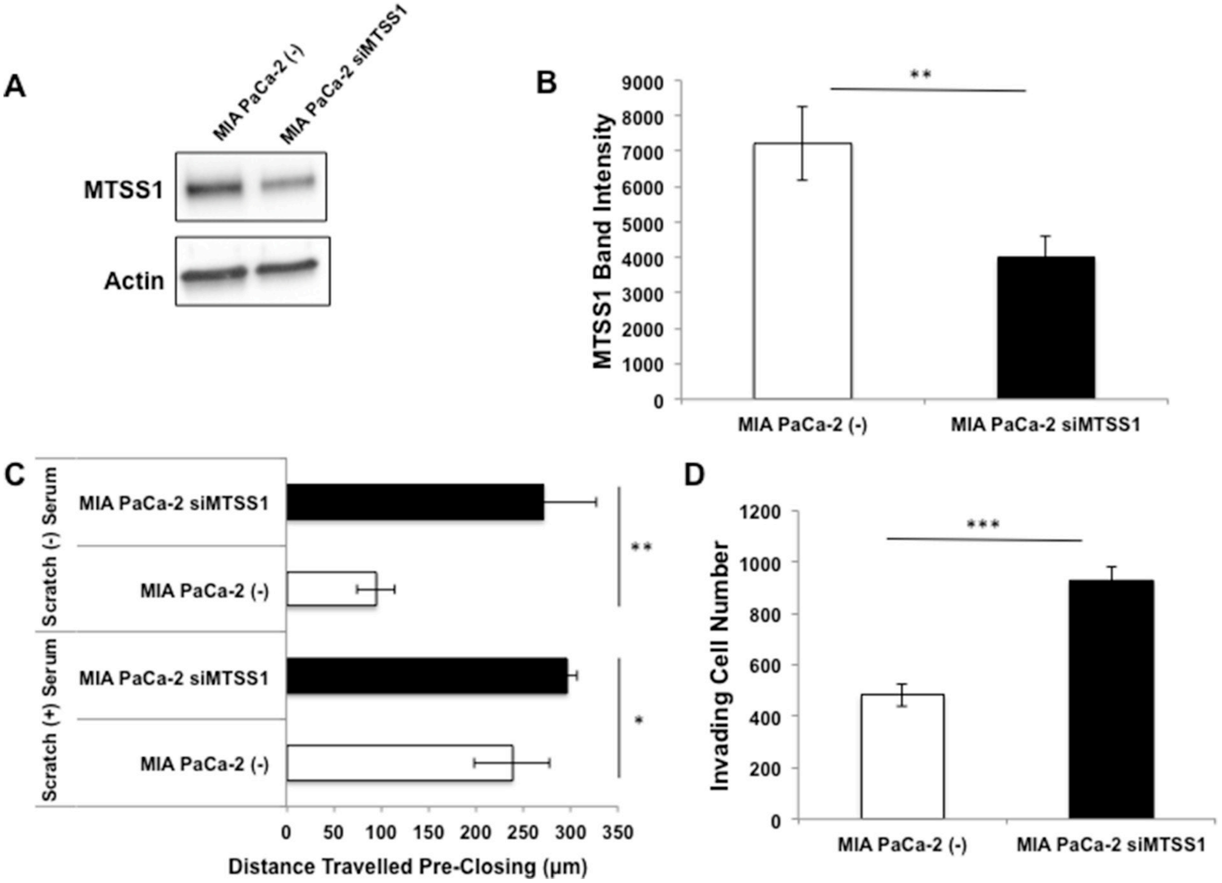

Supplementary Figure 4: Loss of MTSS1 leads to a more invasive and migratory phenotype in additional primary PDAC cell line. (**A**) Western blot confirmation of siMTSS1 knockdown in MIA PaCa -2 cells. (**B**) Densitometry analysis of western blot data of siMTSS1 knockdown in MIA PaCa-2 cells. (**C**) Scratch assays were performed in both serum-containing and serum-free conditions with MIA PaCa-2 (–) and MIA PaCa-2 siMTSS1 cells. (**D**) MIA PaCa-2 (–) and MIA PaCa-2 siMTSS1 cells were plated for a transwell migration assay in the presence of Matrigel, stained with hematoxylin, and counted after 48 hours of incubation. ***p*-value < 0.001.

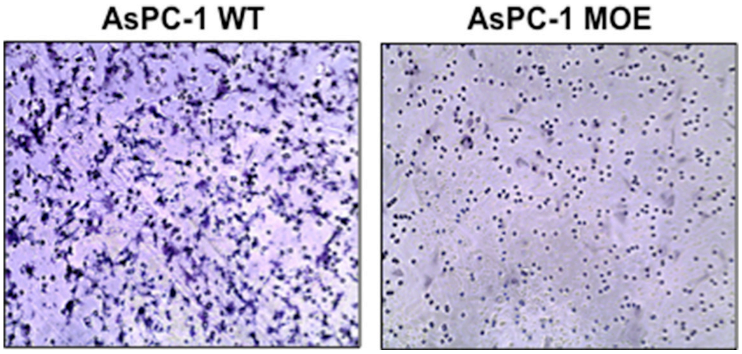

Supplementary Figure 5: Restoration of MTSS1 in metastatic PDAC cell line results in less cell invasion in a transwell assay. Representative images of Matrigel-coated transwell migration assay between AsPC-1 WT cells and AsPC-1 MOE cells. Images were taken at 20× magnification.

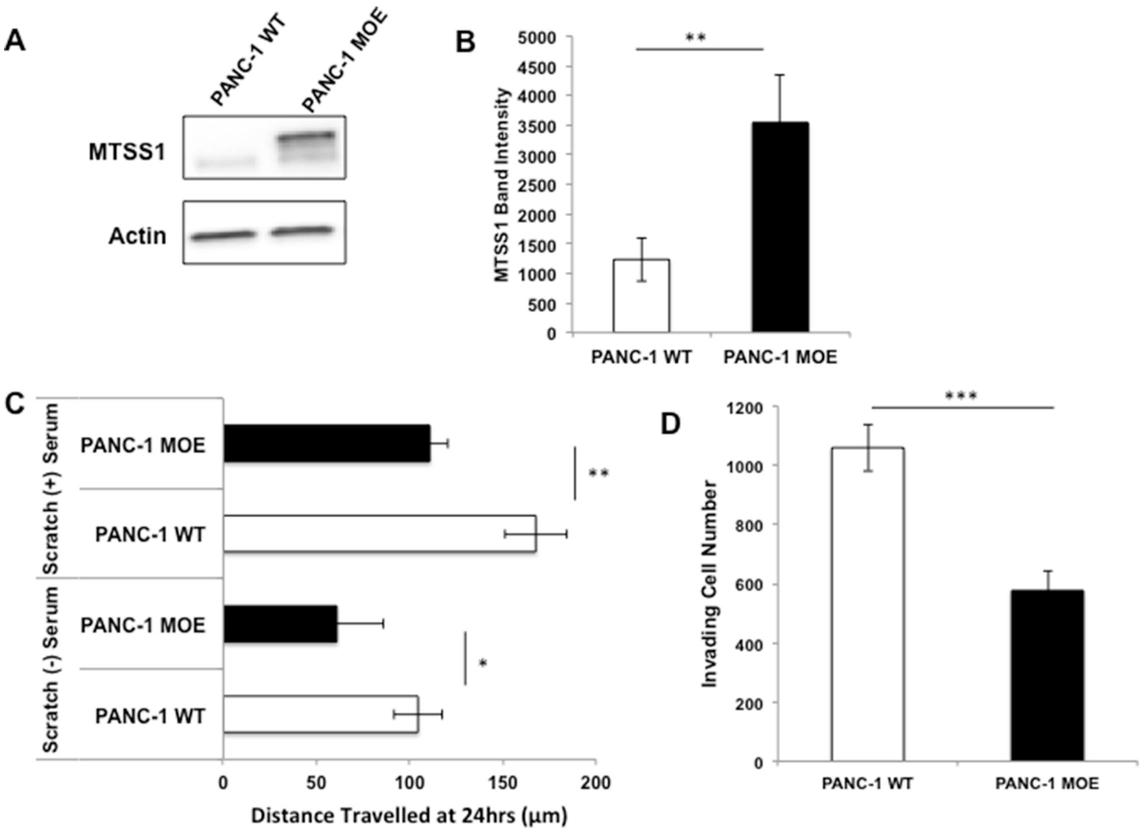

Supplementary Figure 6: Overexpression of MTSS1 leads to a less invasive and migratory phenotype in additional PDAC cell line. (**A**) PANC-1 cells were transduced with MTSS1-Flag plasmid to establish a transient overexpression of MTSS1. (**B**) Densitometry analysis of western blot data of PANC-1 cells transduced with MTSS1-Flag plasmid. (**C**) PANC-1 (–) and PANC-1 MOE cells were plated and scratch assays were performed in both serum-containing and serum-free conditions. (**D**) PANC-1 WT and PANC-1 MOE cells were plated for a transwell migration assay in the presence of Matrigel, stained with hematoxylin, and counted after 48 hours of incubation. *p-value < 0.05, **p-value < 0.001.

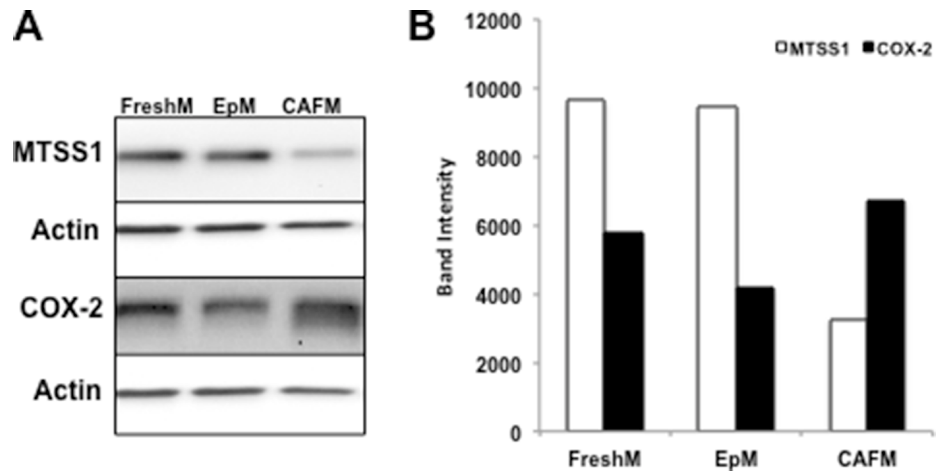

Supplementary Figure 7: Treatment with CAF-conditioned media leads to decrease in MTSS1 and increase in COX-2 expression in PANC-1 cells. (**A**) Western blot analysis of MTSS1 and COX-2 expression in PANC-1 cells treated with either fresh, non-conditioned media (FreshM), EpM, or CAFM for 48 hours before lysate harvest. (**B**) Densitometric quantification of MTSS1 and COX-2 protein expression levels in PANC-1 cells treated with FreshM, EpM, or CAFM.

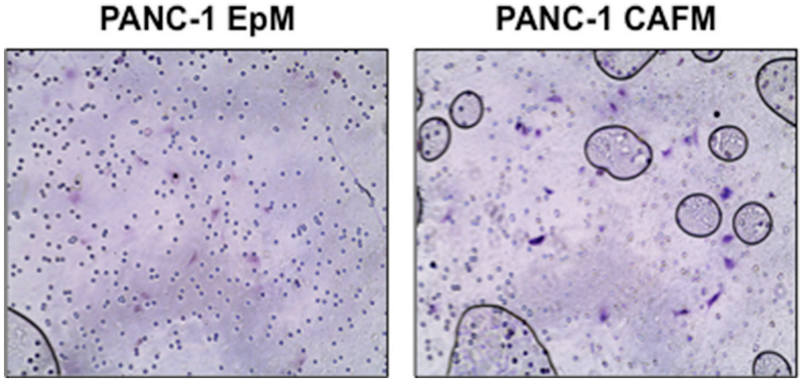

Supplementary Figure 8: Treatment with CAF-conditioned media leads to more cell invasion in a transwell assay. Representative images of Matrigel-coated transwell migration assay between PANC-1 cells treated with either EpM-conditioned media or CAFM-conditioned media. Images were taken at 20× magnification.

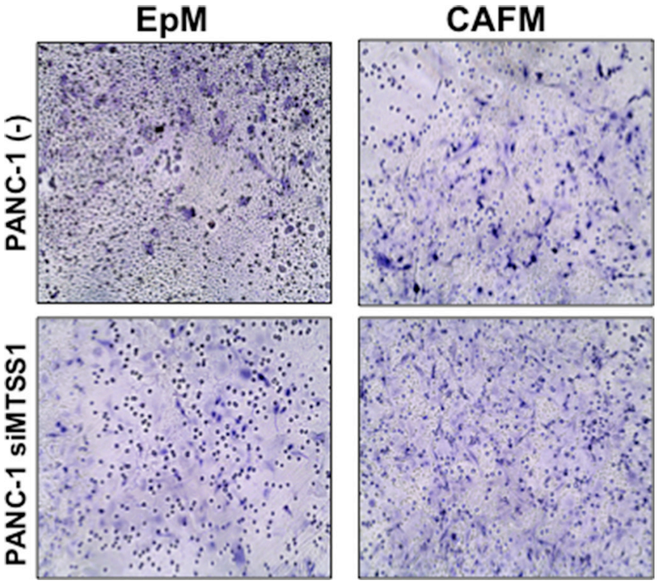

Supplementary Figure 9: Treatment with both MTSS1 siRNA and CAF-conditioned media results in augmented cell invasion in a transwell assay. Representative images of Matrigel-coated transwell migration assay between PANC-1 (–) cells and PANC-1 siMTSS1 cells treated with either EpM-conditioned media or CAFM-conditioned media. Images were taken at 20× magnification.

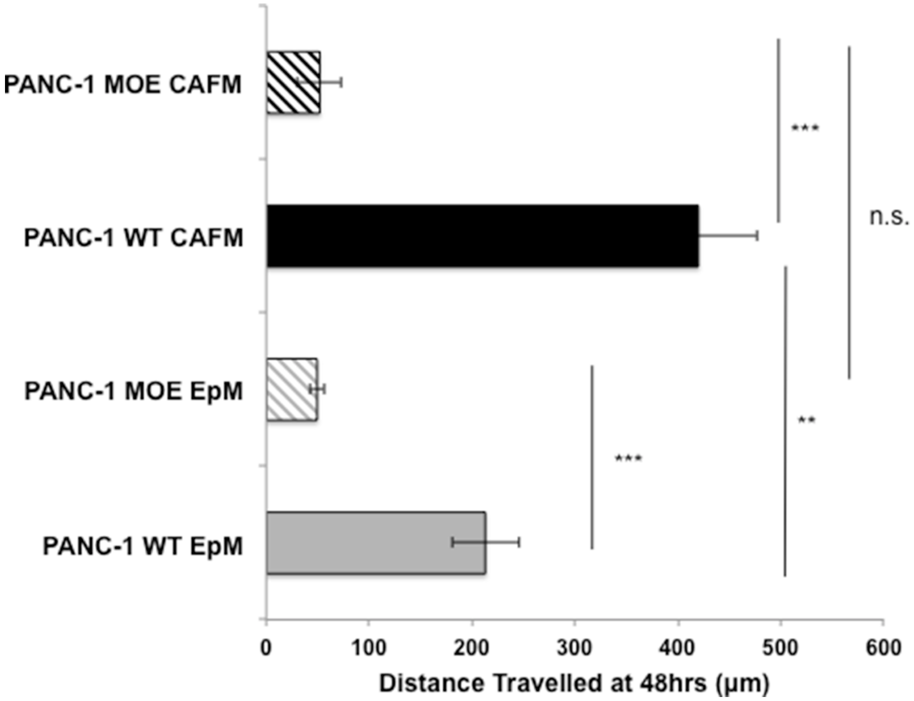

Supplementary Figure 10: Overexpression of MTSS1 results in decreased invasion and migration capabilities in PANC-1 cells regardless of media treatment. PANC-1 WT and MOE cells were treated with either EpM or CAFM and subjected to scratch assay analysis. ***p*-value < 0.001, ****p*-value < 0.0001.

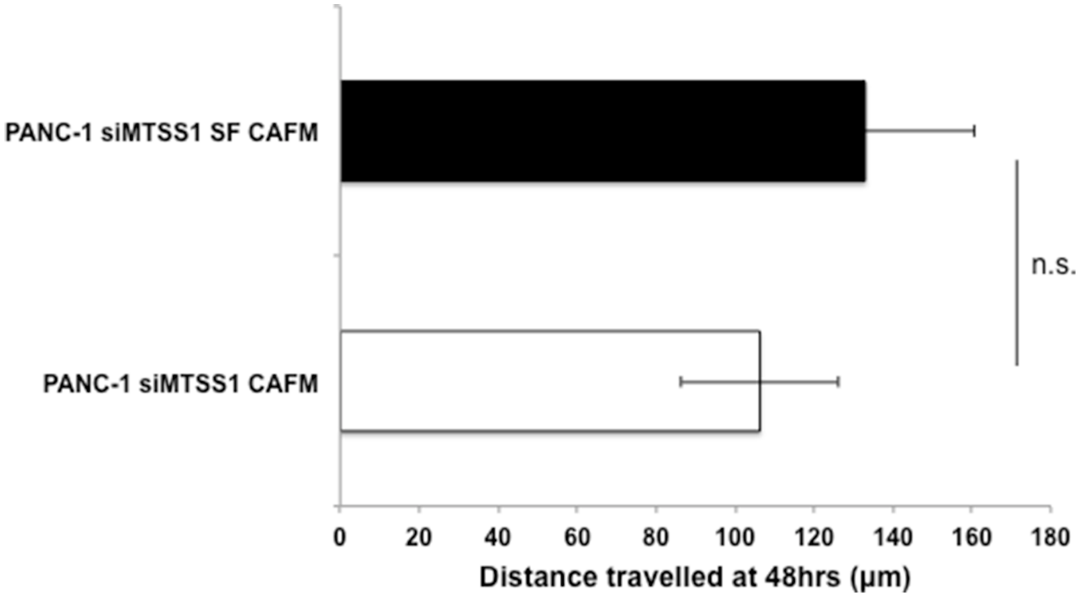

Supplementary Figure 11: Serum-free CAF-conditioned media does not significantly alter the invasion ability of PANC-1 siMTSS1 cells. PANC-1 siMTSS1 cells were treated with either serum-free CAF-conditioned media or complete CAF-conditioned media and subjected to scratch assay analysis.

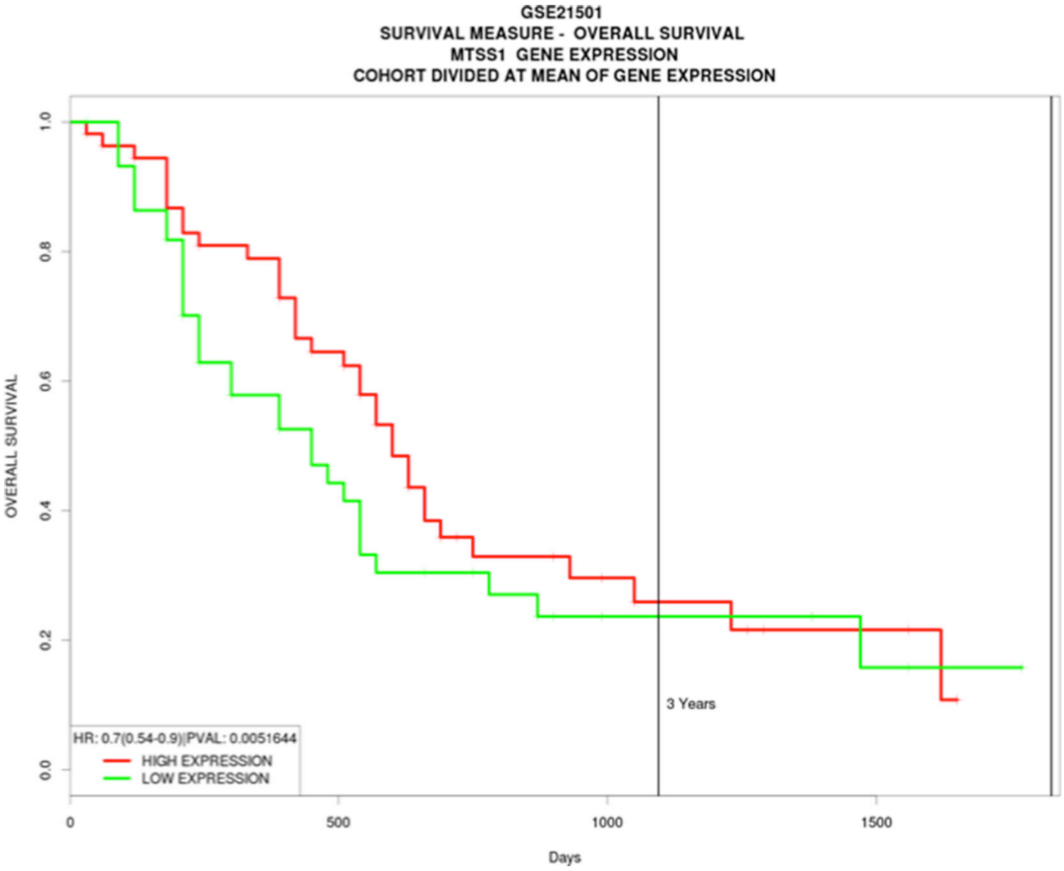

Supplementary Figure 12: Decreased MTSS1 in PDAC patients is an indicator of poor prognosis. Kaplan-Meier survival curve of patient data set GSE21501.

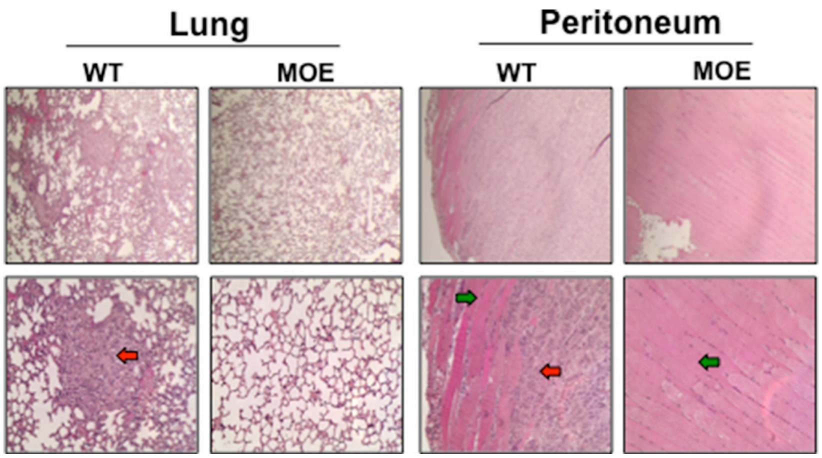

Supplementary Figure 13: Overexpression of MTSS1 in a metastatic cell line results in less tumor cell invasion *in vivo*. Representative H&E images of sections from the lungs and peritoneum of WT or MOE-injected mice at day 51. Red arrows indicate cell invasion found in WT tissue samples. Green arrows indicate normal tissue.

Supplementary Table 1: Differentially Expressed Genes in Kras$^{G12D/+}$;Pten$^{lox/+}$;Cox-2 COE mice compared to baseline

Gene	Fold Change	*p*-value	Gene	Fold Change	*p*-value
Rnase1	−3.57	0.03289	Tmem33	2.00	0.006699
Dnajb6	−2.85	0.023211	Ugp2	2.00	0.015502
Zmynd11	−2.69	0.044665	Siva1	2.04	0.001164
Mtss1	−2.46	0.038618	Wwc1	2.17	0.049824
Tcf25	−2.19	0.002998	Dhx32	2.23	0.003523
Xpot	−2.01	0.036327	Trove2	2.29	0.010156
			Pskh1	2.43	0.012056
			Spg20	2.43	0.009332
			Tmcc3	2.46	0.028791
			Hsd17b11	2.50	0.024350
			Tmtc4	3.14	0.003590
